# Mining the capacity of human-associated microorganisms to trigger rheumatoid arthritis—A systematic immunoinformatics analysis of T cell epitopes

**DOI:** 10.1371/journal.pone.0253918

**Published:** 2021-06-29

**Authors:** Jelena Repac, Marija Mandić, Tanja Lunić, Bojan Božić, Biljana Božić Nedeljković

**Affiliations:** Institute of Physiology and Biochemistry “Ivan Djaja”, Faculty of Biology, University of Belgrade, Belgrade, Serbia; University of Nebraska-Lincoln, UNITED STATES

## Abstract

Autoimmune diseases, often triggered by infection, affect ~5% of the worldwide population. Rheumatoid Arthritis (RA)–a painful condition characterized by the chronic inflammation of joints—comprises up to 20% of known autoimmune pathologies, with the tendency of increasing prevalence. Molecular mimicry is recognized as the leading mechanism underlying infection-mediated autoimmunity, which assumes sequence similarity between microbial and self-peptides driving the activation of autoreactive lymphocytes. T lymphocytes are leading immune cells in the RA-development. Therefore, deeper understanding of the capacity of microorganisms (both pathogens and commensals) to trigger autoreactive T cells is needed, calling for more systematic approaches. In the present study, we address this problem through a comprehensive immunoinformatics analysis of experimentally determined RA-related T cell epitopes against the proteomes of Bacteria, Fungi, and Viruses, to identify the scope of organisms providing homologous antigenic peptide determinants. By this, initial homology screening was complemented with *de novo* T cell epitope prediction and another round of homology search, to enable: *i*) the confirmation of homologous microbial peptides as T cell epitopes based on the predicted binding affinity to RA-related HLA polymorphisms; *ii*) sequence similarity inference for top *de novo* T cell epitope predictions to the RA-related autoantigens to reveal the robustness of RA-triggering capacity for identified (micro/myco)organisms. Our study reveals a much larger repertoire of candidate RA-triggering organisms, than previously recognized, providing insights into the underestimated role of Fungi in autoimmunity and the possibility of a more direct involvement of bacterial commensals in RA-pathology. Finally, our study pinpoints Endoplasmic reticulum chaperone BiP as the most potent (most likely mimicked) RA-related autoantigen, opening an avenue for identifying the most potent autoantigens in a variety of different autoimmune pathologies, with possible implications in the design of next-generation therapeutics aiming to induce self-tolerance by affecting highly reactive autoantigens.

## Introduction

Autoimmune diseases include a broad spectrum of chronic and clinically heterogeneous conditions that affect approximately 5% of the population worldwide, with the tendency of increasing incidence throughout developed countries [1,2]. The complex interplay of host genetics (i.e. polymorphisms of Human Leukocyte Antigen (HLA) gene locus) and environmental factors (i.e. immunization status, infection) is in the base of autoimmune pathologies. Among these, Rheumatoid Arthritis (RA)–a condition characterized by chronic joint inflammation and swelling—represents a considerable health-care burden due to associated prevalence of 0.5%–1% throughout the worldwide population, where only 50% can be connected to familial risk, witnessing of the strong environmental input to the etiology of this disease [3–5].

**Fig 1:**
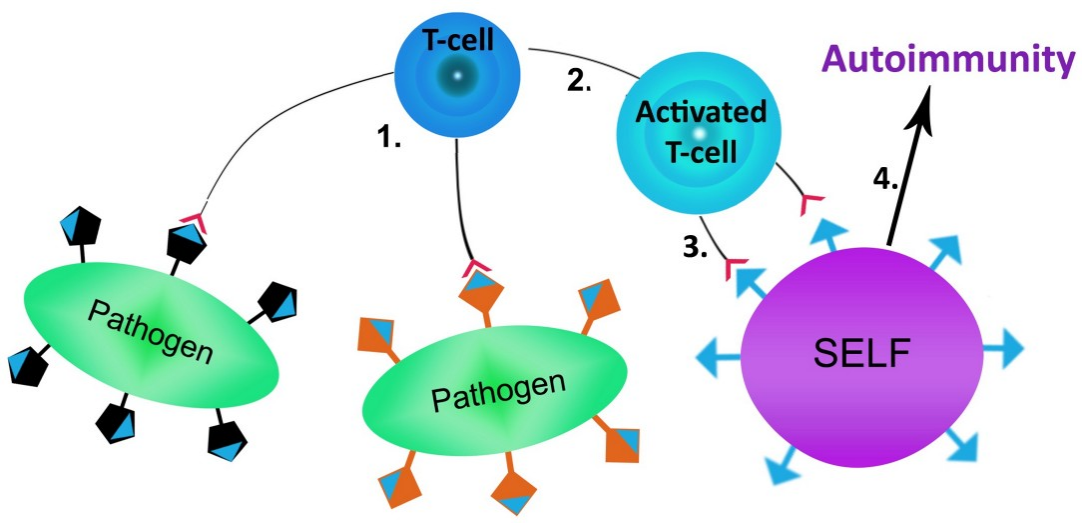
Simplified schema of molecular mimicry. 1) Pathogens are recognized and presented by antigen-presenting cells to pathogen specific T cell; 2) Activation of T cell by recognition of pathogen antigen; 3) Recognition of self-antigen by pathogen activated autoreactive T cell; 4) Triggering of autoimmune process.

One of the most frequent triggers of autoimmunity is infection, whereby molecular mimicry represents a well-recognized and one of the leading underlying mechanisms of infection-mediated autoimmunity [6–8]. Molecular mimicry refers to the similarities between foreign and self-peptides, enabling the activation of autoreactive lymphocytes by foreign-derived peptides in a susceptible individual (Fig 1). Understanding molecular mimicry and its co-factors is a very complex process. Multiple contributing factors such as [9–14]: host genetics; associated gut/oral microbiota; and exposure to other environmental causes (e.g. chemicals) require comprehensive analysis for a more complete insight into the molecular mimicry mechanisms, as well as its impact to autoimmunity.

Currently, four potential modalities of molecular mimicry have been proposed across the literature [15]. Given that molecular mimicry assumes homology between a large number of microbial proteins and proteins from human tissues, identification of novel pathogen species, capable of inducing autoimmune disorders, such as RA, requires a systematic approach. By this, given the immense diversity of the human microbiome [16,17] the possibility that commensal species too, at least under dysbiotic conditions, elicit autoreactive RA-directed lymphocyte responses through molecular mimicry should not be overlooked.

The leading immune cells in the development of RA are T lymphocytes, particularly T helper (Th) 1 and Th17 cells [18,19]. The imbalance of these effector cells and the regulatory lymphocytes represents a main hallmark of RA pathogenesis. Additionally, T-cell receptors (TCR) that recognized foreign antigens display wide specificity and avidity, which play a central role in the loss of self-tolerance [20]. Given that TCR only recognizes antigen peptides within HLA molecules on antigen-presenting cells, HLA molecules comprise a very important governing factor for autoimmunity. The HLA gene locus is highly polymorphic and represents the most investigated genetic factor related to RA [21–23]. In this context, special attention should be focused on identifying as broader as possible scope of potential antigens that trigger autoreactive T cells related to RA. This requires a detailed analysis of the capacity of proteomes from human-associated microorganisms—both pathogenic and commensal—to present within the corresponding HLA molecules T cell epitopes of high enough sequence similarity to RA-related autoantigens.

In the present study, a comprehensive immunoinformatics analysis of the experimentally determined T cell epitopes related to RA was performed. The investigation of the bacterial, fungal, and viral proteomes potential to trigger RA by molecular mimicry was examined, given that each of these groups of organisms harbors human-associated pathogens and commensals. The overarching goal of this *in silico* research was to identify the scope of unrecognized microbial RA-triggers, complemented with the corresponding pool of RA-specific T epitopes, which may elicit autoreactive T cell response for a more robust role in RA pathogenesis.

## Methods

### Sequence datasets

The total of 234 experimentally derived T cell epitopes (S1 Table), related to RA, were downloaded from the IEDB database (Immune Epitope Database and Analysis Resource [24]) on 03/12/2020. Epitopes were retrieved from the IEDB by applying the following search restrictions: in the **Epitope Search Panel** the *Any Epitopes* was selected; in the **Antigen Search Panel** the parameter Organism was set to *Homo sapiens* (ID:9609); in the **Host Search Panel** the *Humans* option was selected; in the **Assay Search Panel** options *Positive Assays Only* and *T cell Assays* were selected, and, finally, in the **Disease Search Panel** the disease *Rheumatoid Arthritis* (ID:DOID:7148) was selected. After removing duplicated sequences that occur in the database due to different posttranslational modifications, the total number of epitopes reduced to 206. Among these, the Epitope 28 could not be related to any antigen provided for RA T cell epitopes in the IEDB database, so we excluded it from further analysis, obtaining the final number of 205 epitopes. All the unique epitope sequences were assigned to the originating antigens (37 in total, S2 Table). The epitopes: 14, 22, 25, 43, 47, 53–59, 63–66, 75, 194, 212, and 233, related to collagen alpha-1(II) chain and corresponding collagen precursors, were re-assigned to the Antigen 10 (Collagen alpha-1(II) chain).

### BLASTp analysis

The blastp (algorithm version: BLASTP 2.11.0+ [25]) search was performed with T cell epitopes for RA, as query sequences, against the following organism groups: Bacteria (taxid: 2), Fungi (taxid: 4751) and Viruses (taxid: 10239). The remaining parameters were set at default values.

The obtained hits were further filtered according to the following criteria: percent identity ≥ 80%, query cover ≥ 80%, no gaps allowed, and e-value < 1. The protein sequences corresponding to the hits obtained by blasting the T cell epitope sequences over Bacteria/Fungi/Viruses were retrieved from the NCBI (https://www.ncbi.nlm.nih.gov/protein). Unique accession numbers for blasted and filtered bacterial, fungal and viral proteins were further used for retrieving the corresponding taxonomy information. Source Organism Lists for retrieved protein hits (BLASTp results) were obtained through the NCBI Entrez System.

### Assigning pathogenicity/commensalism to humans for retrieved organisms

Blasted bacteria, fungi and viruses (from the Source Organism List) were submitted to the NCBI Taxonomy Browser (https://www.ncbi.nlm.nih.gov/Taxonomy/Browser/wwwtax.cgi) to infer the pathogenicity/commensalism to humans. If the information regarding the searched organism host was not available, the extensive literature search was performed instead. In borderline cases, when only rare infections in humans for a given organism had been detected or a given organism had been detected in samples of human origin, without being firmly established as a constituent of human microbiome, a given organism was assigned as putative human pathogen/commensal.

### Mining the literature for association between retrieved organisms and rheumatoid arthritis

To optimize the process of literature search, the association between retrieved organism and RA was initially established at the level of genus, by systematically searching the PubMed database with the following search terms: *rheumatoid arthritis* AND *genus of the given organism* on 21/12/2020. For organisms belonging to the genera retrieved after the initial PubMed search, an in-depth *de novo* search at the species level was performed on 18/01/2021–20/01/2020. By this, careful manual curation of the literature was performed to establish if the association of a given organisms with RA putatively refers to the molecular mimicry as the causative agent of RA. Only these cases were taken into consideration, while cases in literature referring to e.g. infection with a given pathogen due to immunosuppressive therapy after RA were filtered out. Organisms for which association to RA was established were further designated as *PubMed Organisms*, while the remaining ones were designated as *NoPubMed Organisms*.

### T cell epitope prediction

Blasted and filtered FASTA protein sequences (see BLASTp analysis, Methods), originating from the organisms with assigned pathogenicity/commensalism to humans (see Assigning pathogenicity/commensalism to humans for retrieved organisms, Methods), were submitted to the TepiTool on IEDB (version v2.24, [26]) for T cell epitope prediction. By this, only the best scoring protein from the BLAST search (see BLASTp analysis, Methods), in terms of e-value, was submitted to the T cell epitope search for each organism. For bacterial and fungal proteins, the prediction pipeline for the allele MHC class II was used with Human designated as the host. The prediction was performed for a panel of 26 most frequent alleles (providing the human worldwide population coverage > 95%) and applied default settings for a moderate number of peptides (number of overlapping residues in 15mers fixed at 10, with duplicated peptides removed). The currently recommended prediction method by IEDB was used and the threshold set to the top 10% of the predicted peptides (default value). For viral proteins, the prediction was performed for the allele MHC class I, for the panel of 27 most frequent A & B alleles. Default settings for a moderate number of peptides were applied (only 9mers were considered for the prediction with duplicated peptides removed). The currently recommended prediction method by IEDB was used and the threshold set to the top 2% of the predicted peptides (default value). Among the predicted T cell epitopes for each analyzed protein, the candidate epitope(s) showing extensive homology to the corresponding experimental IEDB epitope(s) (S1 Table) were identified and marked red/bold based on the coordinates from the mutual BLASTp alignment (S1–S5 Files).

### Inferring autoreactivity for predicted T cell epitopes through homology search

The potential for triggering an autoimmune response in humans was inferred for the top 0.5% of the predicted T cell epitopes per each protein previously analyzed in TepiTool (corresponding to the best blasted hit for a given human pathogen/commensal). This was conducted through local pairwise sequence alignment of predicted epitopes against the 37 protein sequences of experimentally inferred Human RA Antigens (S2 Table). For the predicted T cell epitopes of bacterial and fungal origin, the sequences associated to those MHC II polymorphisms with no established relation to RA across the literature (DRB5*01:*01*, DQA1*01:01/ DQB1*05:*01* and DQA1*04:01/ DQB1*04:*02* from the panel of 26 most frequently occurring alleles applied in TepiTool), were filtered out from the search as unlikely epitope candidates for RA. Also, the subset of predicted T cell epitopes with substantial sequence homology to the initial pool of experimentally inferred T cell epitopes from IEDB (S1 Table), were filtered out from the search to avoid false positive results.

The first alignment iteration corresponded to pairwise local alignment of selected epitopes (S1–S5 Files) against 37 Human RA Antigen sequences, respectively, through the MATLAB built-in function *localalign* with default parameters (MATHWORKS), returning both optimal and suboptimal alignment results. For each epitope, the best scoring antigen sequence was selected based on the obtained *localalign* score for another alignment round on the Expasy server, through the LALIGN tool with default settings (https://embnet.vital-it.ch/software/LALIGN_form.html, [27]). The predicted T cell epitope queries hitting the corresponding most similar antigen with in LALIGN with the e-value < 0.001 (producing statistically significant alignment) were considered as novel candidates for T cell epitopes, enabling autoimmune/autoreactive responses by molecular mimicry.

### Epitope clustering and sequence logo

For predicted T cell epitopes, originating from bacterial and fungal proteins and with established homology to Antigen 1 (see Inferring autoreactivity for predicted T cell epitopes through homology search, Methods), clustering analysis was performed on the IEDB Analysis Resource section with the Epitope Cluster Analysis tool (http://tools.iedb.org/cluster/, [28]) to infer the level of sequence similarity/identity. The analysis was performed with default parameters, assuming minimum sequence identity threshold of 70% and Cluster-break for clear representative sequence as a clustering method (S6 File). Obtained clusters, comprising minimum five epitope sequences, were further submitted to the enoLOGOS tool [29] to generate the corresponding sequence logos. The analysis was performed with default parameters for input protein sequences and preserved alignment information, obtained from the prior clustering analysis.

**Fig 2:**
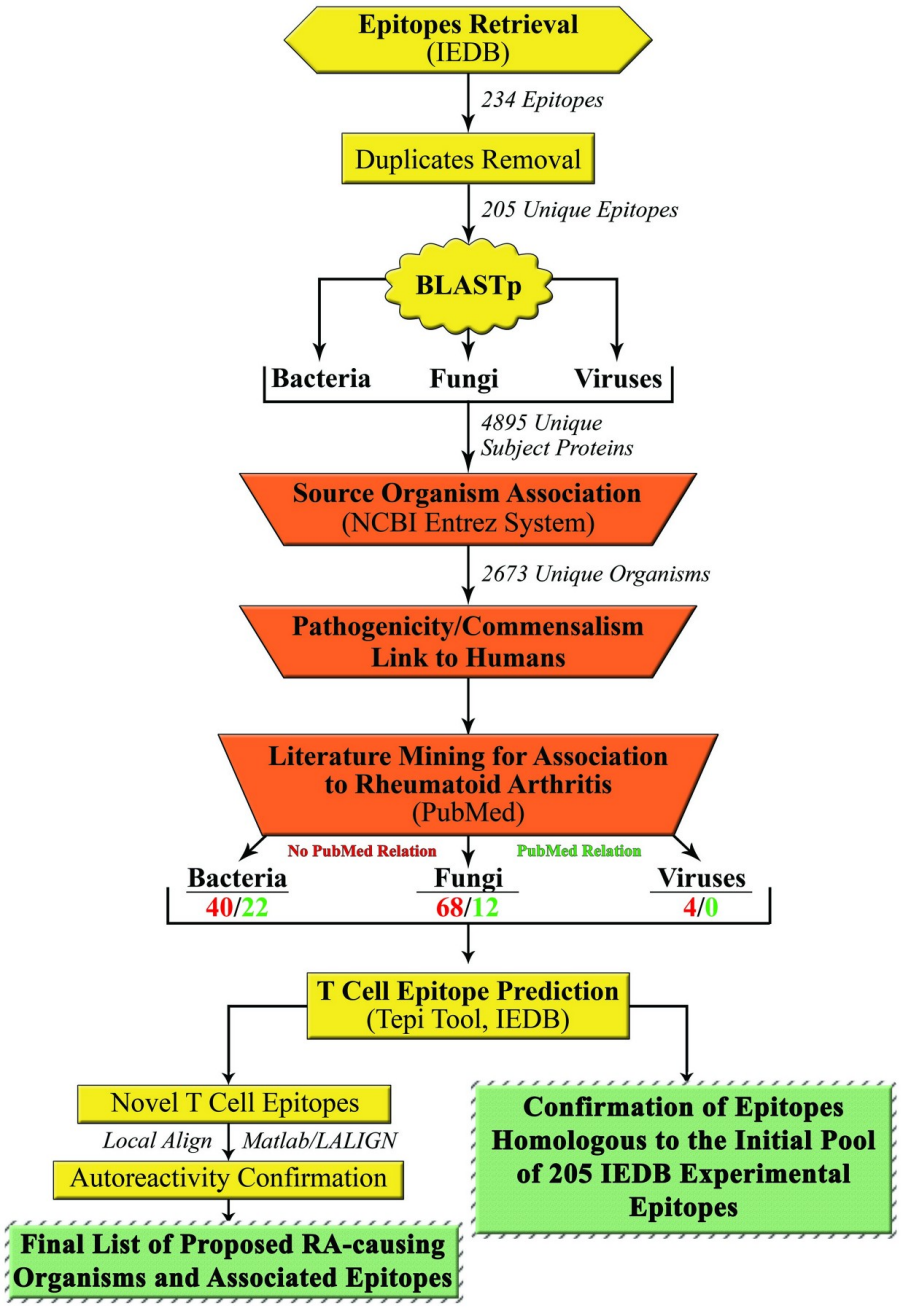
Pipeline for identification of putative RA-triggering organisms together with the corresponding T cell epitopes.

In summary, the pipeline of the proposed research concept is presented on the Fig 2.

## Results

### T cell epitopes for Rheumatoid Arthritis—Searching for homology across human pathogens and commensals

To unravel the full capacity of human-associated microorganisms to putatively trigger RA through molecular mimicry, a comprehensive homology search has been performed. For this, a total of 234 experimentally confirmed T cell epitopes related to RA were obtained from the IEDB database (S1 Table), along with originating 37 Antigen sequences (S2 Table). Among these, a fraction of duplicated epitope sequences occurred, due to having different post-translational modifications, so the total of 205 epitopes with unique sequence specificity was selected for further analysis. All the unique epitope sequences were blasted (BLASTp) separately over Bacteria, Fungi, and Viruses, where the alignments characterized by: percent identity ≥ 80%, query cover ≥ 80%, no gaps, and e-value < 1 were considered as significant hits. Note that the restrictive alignment metrics, in terms of sequence identity, query cover and number of gaps allowed was set according to the linear nature of T cell epitopes and the requirement for as high as possible sequence similarity for assumed epitope cross-reactivity. Otherwise, more liberal e-value threshold was set according to the overall short input sequence(s)/epitopes length.

**Fig 3:**
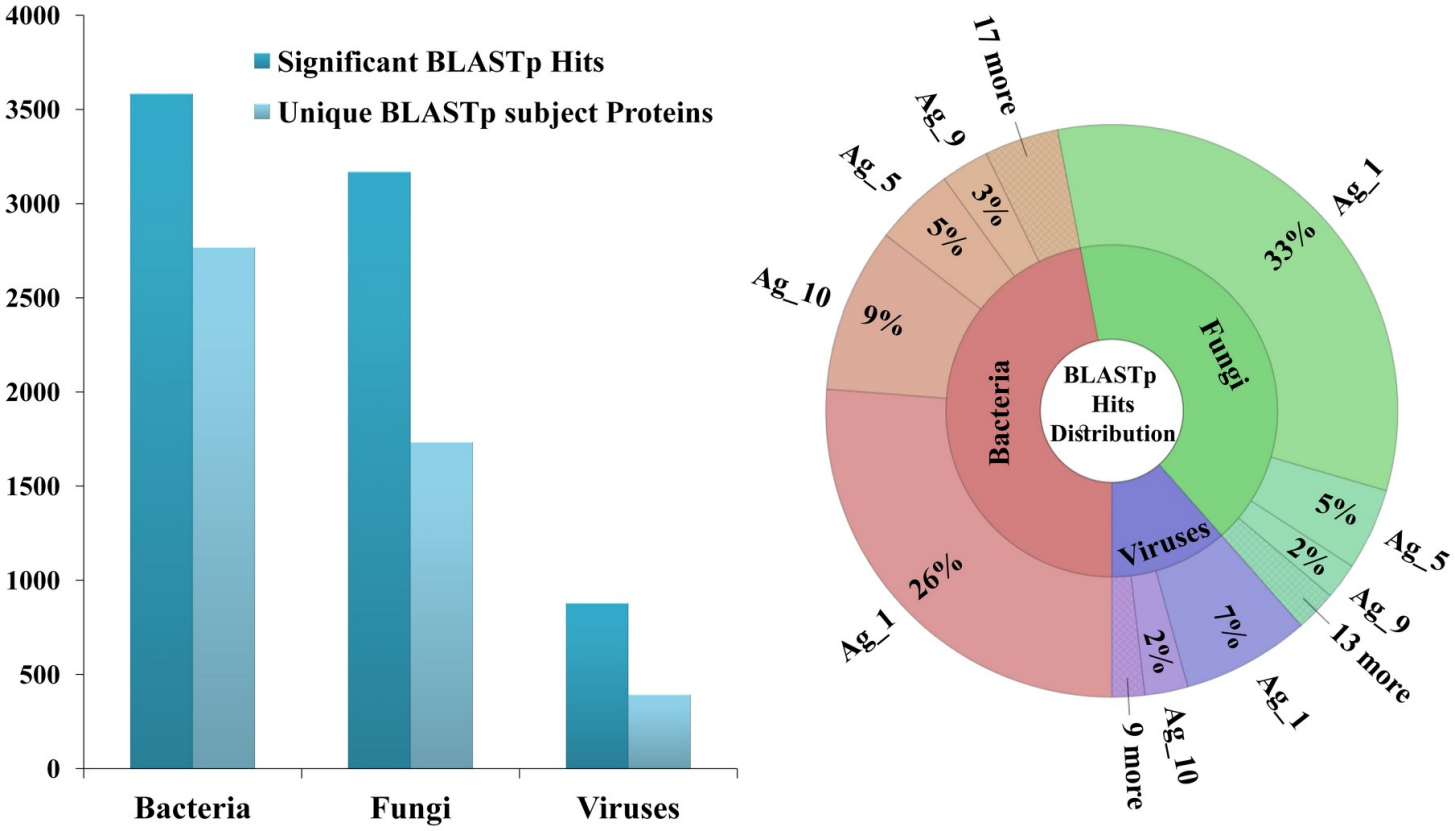
The distribution of all the significant BLASTp hits, together with the proteins they originate from, across Bacteria, Fungi, and Viruses (*left*); The distribution of all the significant BLASTp hits in the context of homologous antigens (the ones associated to the corresponding query experimental RA epitopes) presented for Bacteria, Fungi, and Viruses separately (*right*).

Generally, the majority of significant hits were characterized with e-values < e^–3^ (S4–S8 Tables), even from viral proteins, that display the least homology with their human counterparts. Overall, BLASTp search produced the total of 3583 significant hits from 2768 unique bacterial proteins, 3168 significant hits from 1734 unique fungal proteins, and 878 significant hits from 393 unique viral proteins, respectively (Fig 3-left). The corresponding distribution of obtained hits related to the originating Antigen sequences was presented in Fig 3-right (for absolute numbers see S3 Table). It can be observed that the 2/3 of obtained significant hits aligned with epitopes derived from the Antigen 1, with 33% of these hits originating from fungal, 26% from bacterial and 7% from viral proteins. Interestingly, besides the fact that the Antigen 1 is characterized with the highest number of epitopes overall (42/205), the average number of BLASTp hits *per* epitope in Antigen 1 is consistently very high across bacterial, fungal, and viral proteins, compared to the mean values (BLASTp hits *per* epitope), respectively: 47.6% *vs*. 17.49%, 59.1% *vs*. 17.2% and 13.1% *vs*. 4.2%. Also, approximately 10% of BLASTp hits aligned to the epitopes originating from the Antigens 5 and 10, and another 5% to the epitopes from the Antigen 9, while the distribution of the remaining BLASTp hits showed no additional distinguishable patterns in terms of homologous antigen sequences.

To associate obtained BLASTp hits with the originating organisms, the corresponding unique protein accession numbers were used to retrieve the taxonomy information from the NCBI Entrez System. By this, the total of 2673 different bacterial, fungal and viral species were identified as sources of proteins displaying homology to experimental T cell epitopes for RA (data available upon request). Obtained Source Organism List was then used to further narrow down the search to only Bacteria/Fungi/Viruses with established pathogenicity/commensalism to humans, since only human-associated organisms are able of putatively triggering autoimmune responses. The corresponding association of detected bacterial/fungal/viral species to humans was inferred based on the NCBI Taxonomy Browser, usually providing the information regarding the corresponding host for searched entries. When this was not the case, extensive literature search was performed instead. Obtained lists of organisms with assigned pathogenicity/commensalism, along with the supporting literature information, are available upon request. Out of the 2673 BLAST-retrieved microorganisms, we identified in total 146 as related to humans (the sum of *PubMed* and *NoPubMed* RA-related microorganisms from the Methods Scheme 1).

Obtained comprehensive lists of human pathogens and commensals with proteins showing homology to RA-related T cell epitopes, were further searched across the literature for previously established relation to RA. Organisms with established connection were designated as *PubMed Bacteria* and *PubMed Fungi*, since no association of viruses, obtained through the BLASTp homology search, with RA was detected across the literature. On the other hand, novel candidate organisms, with no literature information tagging them as potential causative agents for RA, were designated as *NoPubMed Bacteria*, *NoPubMed Fungi* and *NoPubMed Viruses*, respectively. Full lists of significant BLASTp hits together with associated alignment metrics, corresponding to designated *PubMed* and *NoPubMed* Bacteria, Fungi, and Viruses was presented in S4–S8 Tables, respectively.

A general trend where *NoPubMed-*designated organisms outnumber groups of organisms with previously established connection to RA (*PubMed*-designated organisms) can be observed. For Bacteria this relation equals to 40 *vs*. 22, for Fungi 68 *vs*. 12, while for Viruses the only detectable category was *NoPubMed* with 4 viral representatives. This marked difference implies the existence of a much larger repertoire of organisms able to convey signals/information crucial for triggering RA-related autoimmune response in humans, than previously acknowledged. On the other side, it also brings out the need for systematically investigating the capacity of human-associated microorganisms to induce other autoimmune conditions, where molecular mimicry has previously also been implicated as one of the important prerequisites for the disease onset. Interestingly, the detected difference in the number of *NoPubMed vs*. *PubMed* organisms is quite large for Fungi, implicating that this group of organisms has been neglected over the years in the context of exploring causative agents for autoimmune diseases. On the other side, much closer phylogenetic relation of Fungi to humans, compared to Bacteria, certainly implies larger inherent capacity of contributing to the onset of autoimmune conditions through molecular mimicry.

Although being highly restrictive in terms of applied parameters, our search was able to retrieve all the major players among the Bacteria, such as *Proteus mirabilis*, *Mycobacterium tuberculosis*, *Escherichia coli*, and *Prevotella* species (S5 Table), that were previously widely acknowledged across the literature as important contributors in the etiology of RA. Similarly, among fungal species, our search successfully retrieved numerous *Candida albicans* strains. Finally, a number of different commensal species, originating both from the gut and oral microbiome, like *Bacteroides fragilis*, *Collinsella aerofaciens*, *Capnocytophaga gingivalis*, and *Prevotella copri* have been detected through our search. For all these organisms, the connection with RA and other autoimmune conditions has been previously established.

### T cell epitopes prediction—Mining BLASTp hits homologous to IEDB experimental T cell epitopes and novel epitopes prediction

To further explore the capacity of detected organisms for triggering RA through molecular mimicry, T cell epitope prediction with the TepiTool on IEDB server has been performed. Note that for organisms with more than one BLASTp-retrieved protein, only the one harboring hits with the lowest e-values was selected for the subsequent search. This ensures the prediction of T cell epitopes with the highest potential of raising autoreactive responses in humans. All the results of the analysis were presented in S1–S5 Files (the first sheet, named: Predicted_T_Cell_Epitopes), for *NoPubMed Bacteria*, *PubMed Bacteria*, *NoPubMed Fungi*, *PubMed Fungi*, and *NoPubMed Viruses*, respectively.

Based on the coordinates in originating protein sequence, predicted T cell epitopes can be associated to corresponding BLASTp hits. This has been performed in order to trace back the BLASTp hits—the regions of homology in blasted proteins with the pool of initial IEDB experimental T cell epitopes—and confirm them as true T cell epitope candidates from the corresponding bacterial/fungal/viral species. For bacterial and fungal proteins, T cell epitope prediction was performed for MHC class II molecules, for the panel of 26 most common HLA alleles in human population. Note that only those predictions, associated to HLA alleles with established connection to RA across the literature (S9 Table), were considered as true epitope candidates (predictions associated to HLA alleles DRB5*01:*01*, DQA1*01:01/DQB1*05:*01*, and DQA1*04:01/DQB1*04:*02* were excluded from further analysis). Due to intracellular pathway of epitope generation, T cell epitope prediction in viral proteins was performed for MHC class I molecules. For MHC class I gene locus polymorphisms, no clear association to RA was available across the literature, so that the set of forbidden HLA alleles for MHC class I prediction could not be defined.

Among the predicted epitopes, the ones corresponding to retrieved BLASTp hits, that is, to experimental IEDB T cell epitopes, were colored red/bold. In cases when only partial overlap of predicted epitopes with the BLASTp hit for the corresponding protein/species could be detected, those predictions were colored green/bold. Note here that the prediction method applied generated epitopes consistent in length, while in the pool of experimental IEDB epitopes and obtained BLASTp hits, sequences of differing length could be found. Therefore, the exact coordinate match between the predicted epitopes and BLASTp hits could not always be expected. Accordingly, if the BLASTp hit was contained within the predicted epitope with almost entire length (leaving no more than 3 AA overhead), this was considered also as a true match between the two sequences. For MHC class II predictions, the top 1% of the predicted epitopes was bolded also to highlight the best scoring sequence peptides (cutoff for the prediction was 10%), that is, the ones with the highest predicted affinity for binding to MHC class II alleles.

A general trend across all the investigated groups of organisms could be observed showing that the experimental T cell epitopes, that is, BLASTp hits, could be recovered in the majority of organisms among the predicted T cell epitopes, in the corresponding searched proteins. For *NoPubMed Bacteria*, the number of unrecovered BLASTp hits equals to 8 out of 40, whereby in 4 from the corresponding 8 organisms a partial match between the BLASTp hits and some of the predicted epitopes could be found (S1 File). For *PubMed Bacteria*, the number of unrecovered BLASTp hits is somewhat higher, equaling 10 out of 22, whereby in 2 from the corresponding 10 organisms, a partial match could be detected (S2 File). Interestingly, in *P*. *mirabilis*, which represents a bacterium well acknowledged across the literature as a proposed triggering agent for RA, only a partial match with the pool of experimental IEDB epitopes could be found, implying that a fraction of epitopes important for the etiology of this autoimmune condition seems to still remain below the radar of experimental detection. Contrary to this, in *M*. *tuberculosis* and *E*. *coli* a large number of the best scoring BLASTp hits could be recovered among the predicted epitopes. In *E*. *coli*, for example, the best scoring T cell epitope prediction (falling into 0.02% of the peptides displaying highest affinity towards MHC class II molecules) matches one of the obtained BLASTp hits. The ranking of the remaining goes from: 1.5%, 2.5%, 4.6%, 6.9%, and 7.8% up to 8.7%. Similarly, in *M*. *tuberculosis* the best scoring predicted T cell epitope (falling into 0.38%) corresponds to the recovered BLASTp hit, while the ranking of the remaining ones goes from: 0.64%, 0.67%, 0.9%, 2.1%, 2.5%, 3.4%, 3.5%, 3.7%, 4.2%, 4.4%, 4.5%, 4.7%, 4.8%, 4.9%, 5.7%, 5.9%, 7.1%, 7.5%, 7.6%, 7.7%, 8%, 8.1%, 8.35%, 8.6%, 8.7%, 8.8% up to 9.6%.

In the group of *NoPubMed Fungi* only for 9 out of 68 organisms BLASTp hits could not be recovered, whereby for 7 organisms from the corresponding 9 partial matches with predicted T cell epitopes could be detected (S3 File). For *PubMed Fungi*, in 2 out of 12 organisms even partial BLASTp matches could not be detected (S4 File). This does not relate to detected *C*. *albicans* strains, where multiple BLASTp hits could be recovered, resembling the already observed trend in top *PubMed* bacterial triggering agents of RA. Interestingly though, for Fungi much larger number of predicted T cell epitopes overall could be observed, due to closer phylogenetic relation to humans, implying higher potential immunogenicity of their proteins. Finally, when it comes to Viruses, in all 4 representatives matches between obtained BLASTp hits and predicted T cell epitopes for MHC class I molecules could be detected (S5 File). However, due to the lack of relatedness between MHC class I alleles to RA across the literature, these results should be considered with somewhat more caution, as compared to the quite convincing results obtained in the groups of Bacteria and Fungi. A comprehensive list of all the organisms designated as putative triggering agents of RA was presented below, in the Table 1. This list includes both previously known examples from the literature, recovered through our methodology/search, and also novel candidate organisms, previously unrelated to RA etiology.

**Table 1:** A comprehensive list of RA-triggering organisms obtained through presented analysis, comprising both the organisms previously related to RA (confirmed examples from the Literature) and novel candidates.

Bacteria		Fungi		
Putative	Literature	Putative 1/2	Putative 2/2	Literature
***Acetobacter indonesiensis***	*Bacteroides fragilis*	*Absidia glauca*	*Macrophomina phaseolina MS6*	*Candida albicans 12C*
***Acidopropionibacterium timonense***	*Campylobacter jejuni*	*Absidia repens*	*Metarhizium anisopliae BRIP53293*	*Candida albicans 19F*
***Bdellovibrio bacteriovorus***	*Collinsella aerofaciens*	*Apiotrichum porosum*	*Metarhizium brunneum ARSEF3297*	*Candida albicans Ca6*
***Cellulomonas hominis***	*Collinsella sp An271*	*Apophysomyces ossiformis*	*Metarhizium brunneum*	*Candida albicans*
***Gemmata obscuriglobus***	*Escherichia coli*	*Aureobasidium melanogenum CBS11037*	*Metarhizium guizhouense ARSEF977*	*Candida albicans P34048*
***Hazenella coriacea***	*Klebsiella pneumoniae*	*Aureobasidium pullulans EXF150*	*Metarhizium robertsii ARSEF23*	*Candida albicans P37005*
***Laceyella sacchari***	*Mycobacterium tuberculosis*	*Aureobasidium pullulans*	*Metarhizium robertsii*	*Candida albicans P57072*
***Lactiplantibacillus plantarum***	*Nocardia farcinica*	*Blastomyces gilchristii SLH14081*	*Microsporum canis CBS113480*	*Candida albicans P75063*
***Mageeibacillus indolicus***	*Prevotella copri*	*Blastomyces parvus*	*Millerozym farinosa CBS7064*	*Candida albicans SC5314*
***Microbacterium arborescens***	*Prevotella sp CAG255*	*Blastomyces percursus*	*Moesziomyces antarcticus T34*	*Candida albicans WO-1*
***Microcystis aeruginosa***	*Prevotella sp KH2C16*	*Byssochlamys spectabilis No5*	*Moesziomyces antarcticus*	
***Microcystis aeruginosa***	*Prevotella sp oral taxon 820*	*Byssochlamys spectabilis*	*Moesziomyces aphidis DSM70725*	
***Moorea producens***	*Proteus mirabilis*	*Cladophialophora bantiana CBS 173*.*52*	*Nannizzia gypsea CBS118893*	
***Negativicoccus succinicivorans DORA***	*Ruthenibacterium lactatiformans*	*Cladophialophora carrionii CBS16054*	*Paxillus involutus ATCC200175*	
***Negativicoccus succinicivorans***	*Streptococcus pyogenes*	*Cladophialophora immunda*	*Phialemoniopsis curvata*	
***Nodularia spumigena***		*Cladosporium cladosporioides*	*Piedraia hortae CBS480*.*64*	
***Nosocomiicoccus massiliensis***		*Claviceps purpurea*	*Purpureocillium lilacinum*	
***Paraclostridium dentum***		*Colletotrichum tofieldiae*	*Rhinocladiella mackenziei CBS650*.*93*	
***Parascardovia denticolens***		*Conidiobolus coronatus*	*Rhizoctonia solani*	
***Pelomonas puraquae***		*Cyberlindnera fabianii*	*Rhizoctoni solani AG-8 WAC10335*	
***Pelomonas saccharophila***		*Cyphellophora europaea CBS101466*	*Rhizoctonia solani 123E*	
***Peptidiphaga gingivicola***		*Emergomyces pasteurianus Ep9510*	*Rhizoctonia solani AG-1 IA*	
***Planktothrix agardhii***		*Emmonsia crescens*	*Rhizoctonia solani AG-1 IB*	
***Prevotella shahii***		*Emmonsia crescens UAMH3008*	*Rhizoctonia solani AG-3 Rhs1AP*	
***Risungbinella massilensis***		*Emmonsia sp CAC2015a*	*Russula emetica*	
***Scardovia inopinata***		*Galerina marginata CBS339*.*88*	*Sporidiobolus salmonicolor*	
***Scardovia wiggsiae***		*Hortaea werneckii*	*Syncephalastrum racemosum*	
***Sphingobium yanoikuyae***		*Kwoniella pini CBS10737*	*Thermothelomyces thermophilus ATCC 42464*	
***Thermoacttinomyces vulgaris***		*Lasiodiplodia theobromae*	*Verruconis gallopava*	
		*Lichtheimia corymbifera JMRCFSU9682*	*Wallemia mellicola CBS 633*.*66*	
		*Lichtheimia ramosa*	*Yarrowia lipolytica CLIB122*	
		*Lodderomyces elongisporus NRRL YB-4239*	*Yarrowia lipolytica*	

### Inferring the capacity for raising autoreactive responses in humans for newly predicted T cell epitopes

To further explore the capacity of organisms from the previously proposed list (Table 1) to trigger RA through molecular mimicry, a panel of the best scoring novel T cell epitope predictions was chosen for establishing sequence similarity to 37 Antigen sequences related to this autoimmune disease. Novel T cell epitope predictions from bacterial and fungal proteins (not containing epitopes corresponding to BLASTp hits), designated as the top 0.5% sequences in terms of predicted binding affinity for MHC class II molecules (when available), were submitted to the search, due to highest chances of being captured and processed into true epitopes when found in human cells. Among the viral T cell epitopes, those sequences scoring better than the ones corresponding to BLASTp hits, in terms of predicted binding affinity for MHC class I molecules, were selected for the subsequent analysis.

The first round of search was implemented in Matlab, through local pairwise sequence alignment between the corresponding T cell epitope predictions against each of the 37 Antigen sequences. For each T cell epitope prediction, the Antigen sequence producing the highest alignment score in Matlab was selected for confirmation of mutual sequence similarity through *de novo* local pairwise alignment in LALIGN, which provides statistical significance (e-value) for produced alignments. When more than one Antigen sequence aligns in Matlab with the same highest score to a given epitope, all of these were further submitted for a novel round of alignment in LALIGN with the corresponding epitope. LALIGN alignments characterized with e-values < e^–3^ were considered a confirmation of the previously established sequence similarity in Matlab for a given T cell epitope–Antigen pair. That is, T cell epitope predictions producing statistically significant alignments with any of the 37 Antigen sequences were considered as candidates for raising autoreactive responses in humans, putatively leading to the RA pathology. The alignment results were presented in S1–S5 Files (the second sheet, named: Lalign_Autoreactivity_Crosscheck), for *NoPubMed Bacteria*, *PubMed Bacteria*, *NoPubMed Fungi*, *PubMed Fungi*, and *NoPubMed Viruses*, respectively.

A general trend across all the investigated groups of organisms shows that newly predicted T cell epitopes in the majority of explored cases/organisms display significant sequence similarity against Antigen sequence(s) related to Rheumatoid Arhtritis, implying even larger capacity for these organisms to provide RA-triggers in humans, beyond previously established homology to a given experimental T cell epitope through BLASTp and TepiTool analyses. In the group of *NoPubMed Bacteria*, in only 6 out of 36 bacterial species/strains significant alignments of novel T cell epitopes with RA Antigens were not obtained. In the group of *PubMed Bacteria*, the same result occurs in 4 out of 15 organisms, while in the groups of *NoPubMed Fungi* and *PubMed Fungi* the numbers go even smaller, pointing to only 2 out of 66 and 2 out of 12 organisms, respectively, with no significant sequence similarity of the predicted best scoring T cell epitopes to any of the 37 RA Antigens. For *NoPubMed Viruses*, on the other hand, sequence similarity between the chosen predicted T cell epitopes and RA Antigens could not be confirmed through LALIGN in a vast majority of cases, due to too short input sequence length (LALIGN allows the alignment of only the peptides ≥ 10 AA). However, even under these restrictions, significant alignments were produced for some of the predicted T cell epitopes from 3 out of 4 analyzed viruses, though with borderline e-values, up to e^–4^. Contrary to this, significant LALIGN alignments of bacterial and fungal T cell epitope predictions with RA Antigens, display more convincing e-values, predominantly in the span between e^–5^ and e^–10^, with the best/lowest e-value equaling e^–11^.

**Fig 4:**
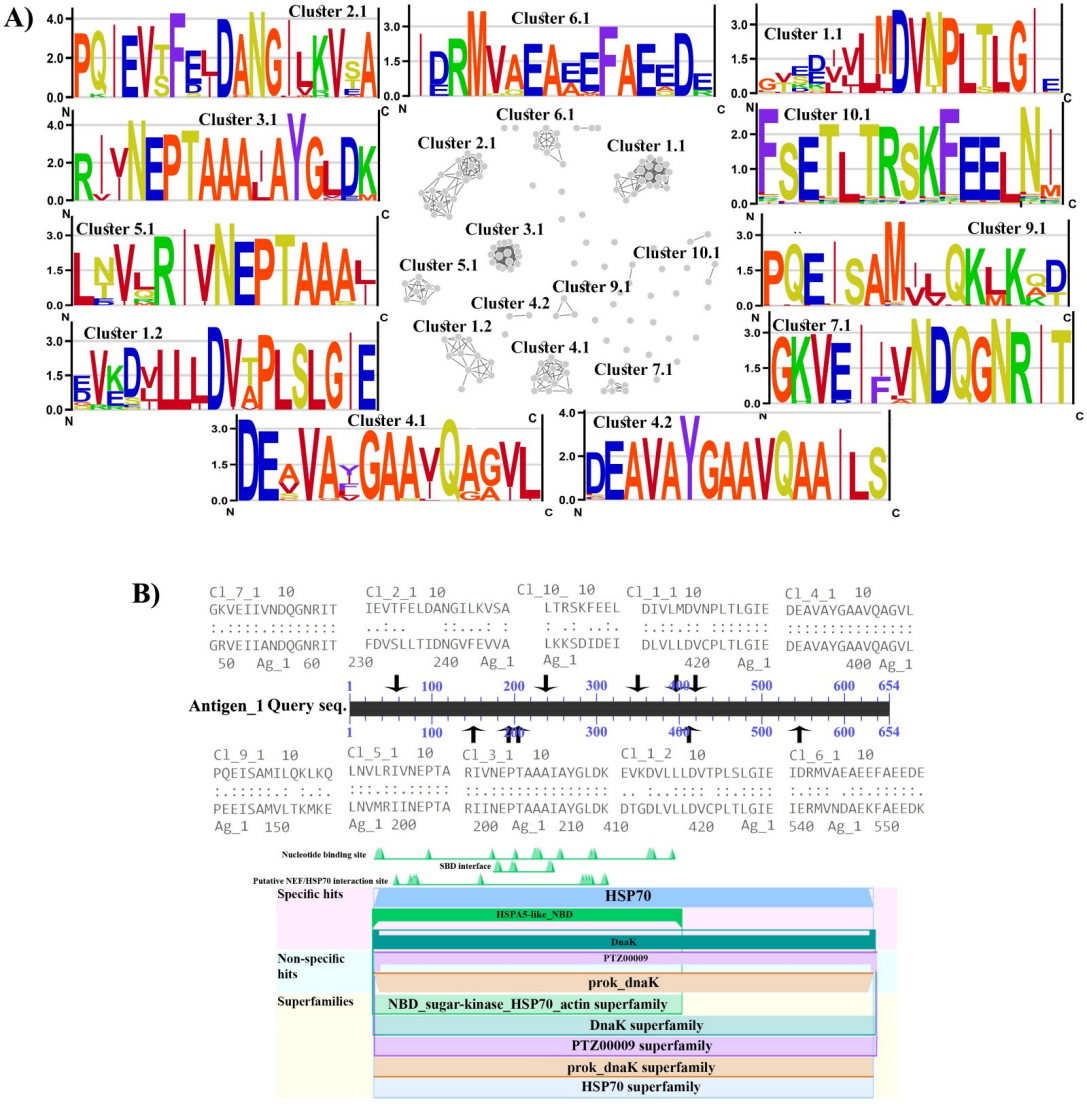
A) Cluster representation for bacterial and fungal predicted T cell epitopes with significant sequence similarity to the Antigen 1, together with the corresponding sequence logos for the largest epitope clusters; B) Mapping of the T cell epitope clusters to the Antigen 1 together with its corresponding conserved domains positions.

The largest fraction of T cell epitope predictions with confirmed sequence similarity to human RA Antigens across all the investigated groups of organisms aligns with the Antigen 1, corresponding to the Endoplasmic reticulum chaperone BiP from the highly conserved protein family Heat Shock protein 70 (HSP70). A comprehensive list of both the experimental and the predicted T cell epitopes corresponding to this Antigen 1 was presented in the S10 Table. Sequence similarity for a large fraction of these epitopes can easily be noticed, pointing probably to the regions of Antigen 1 with the highest level of conservation, as would be expected to underlie significant alignments between the sequences phylogenetically very distantly related, such as human and bacterial protein sequences. To explore further this apparent sequence similarity, predicted T cell epitope sequences related to the Antigen 1 were submitted for clustering analysis, with the results presented in Fig 4A. Here, 11 clusters containing at least 5 different T cell epitope sequences can be observed, displaying substantial sequence conservation across the entire alignment length, as evidenced on the corresponding ENOLOGOS representations, surrounding the clusters. In the Fig 4B mapping of the obtained clusters onto the Antigen 1 sequence was shown, together with the prediction of conserved domains in this protein. Here, it can be observed that all the obtained highly conserved consensus epitope sequences/clusters fall indeed within the predicted HSP70 protein family domain, which, however, spans almost the entire protein length. Besides with the Antigen 1, significant alignments between the predicted T cell epitopes and RA Antigens were also obtained for the Antigen 2, Antigen 5, Antigen 11, Antigen 12, Antigen 19, Antigen 21, Antigen 31, though usually in much smaller copy numbers and borderline e-values from e^–3^ and e^–4^. To a certain extent, Antigens 5 and 11, corresponding to the Alpha-enolase and MHC class IB, respectively; represent exceptions with the alignments characterized by e-values in the similar range as the alignments corresponding to the Antigen 1.

## Discussion

In the present study, an in-depth immunoinformatics analysis, which explores the capacity of human-associated microbes to trigger RA through molecular mimicry, was performed. Results of this study reveal a wide spectrum of species able to elicit RA-specific autoreactive T cells. To the best of our knowledge, this is the first study that systematically addresses the specificity of autoreactive T cells (i.e. their corresponding epitopes), which are central to the RA etiopathogenesis, especially for initiating cascades of events. Although B cells also contribute to the RA pathology, these cells are not crucial for the triggering of autoimmunity, but rather in further phases of the disease. Also, B cells are fully activated to produce autoantibodies by additional input from CD4^+^ T cells, whose activity is captured in the presented analysis by T cell epitope prediction for MHC II molecules [30–32].

The number of candidate (human-associated) RA-triggering organisms identified in this study corresponds to ~5% of the total number of blasted microorganisms. We hypothesize that larger fraction would probably be hazardous from the aspect of increased autoimmunity incidence in predisposed individuals. However, it seems that not all candidate RA-triggers harbor the same-level potential of causing RA-autoimmunity. Namely, the number of blasted sequences in different *E*. *coli* or *M*. *tuberculosis* strains equals ~100, while at the same time microbes harboring only one or several blasted hits constitute the majority among detected organisms. In contrast to this, a large fraction of proposed RA-triggering candidates refers to the microbes previously uncharacterized as contributing factors to develop this autoimmune condition. However, major players, which have been identified in the literature as the most probable RA-triggers, such as *P*. *mirabilis* [33–39] and *M*. *tuberculosis* [40–45], also found their place in the proposed lists of candidate species (Table 1), corroborating the applied search methodology. Additionally, a set of predicted T cell epitopes, with significant sequence similarity to known RA autoantigens, witnesses of a more robust capacity of proposed microbial triggers to activate autoreactive T cells and, therefore, contribute to the RA development.

Among the proposed RA-triggering candidates, arrays of well-recognized and also rarely-occurring pathogens stand in parallel with arrays of opportunistic pathogens and commensal species. This suggests that the constituents of human microbiome might take a more direct involvement in the pathogenesis of this highly incident autoimmune disease, beyond indirect local (i.e. increased intestinal barrier permeability for pathogenic species) or systemic effects (i.e. the state of low-level chronic inflammation), favored usually under dysbiotic conditions [4,46–48]. Related to this, constituents of both gut and oral microbiota, such as *B*. *fragilis*, different *Prevotella* strains and *C*. *gingivalis*, were confirmed as candidate RA-triggers in this study, being in line with recent literature data that connects RA to the perturbed oral and gut microbiome [49–51]. Moreover, given that colonization by opportunistic pathogens assumes pre-existing perturbations in immune system functioning [52–54], these conditions might also provide a fruitful substrate for increasing susceptibility to the autoimmune phenomena, through molecular mimicry mechanism. Also, in this study a number of commensal bacteria, previously unrelated to the development of RA, such as *A*. *timonense*, *B*. *bacteriovorus*, *P*. *puraquae*, *P*. *saccharophila*, *P*. *shahii*, and *R*. *massiliensis*, were proposed as possible RA-triggers. As previously stated, dysbalanced microbiota, primarily caused by altered (mal)nutritional habits throughout Western civilization countries could be related to the RA-triggering, through molecular mimicry mechanism. This, therefore, calls for more intensive research efforts to establish all the missing links on the axis nutrition–microbiota–increased incidence of RA in Western civilization.

A considerable RA-triggering capacity was also reported for Fungi in this study. Although regularly populate human microbiome (mycobiome) or represent threatening pathogens, these organisms are generally overlooked as potential triggers for RA-development. However, a much closer phylogenetic relation of Fungi to humans, compared to Bacteria and Viruses, certainly implies a larger inherent capacity for molecular mimicry, due to the overall broader scope of shared homologous peptides with human proteins. This is well illustrated by the fact that the number of newly proposed T cell epitopes for Fungi that significantly align to RA-related autoantigens equals ~500 *vs*. ~200 of predicted RA-specific T cell epitopes reported in Bacteria. In this context, somewhat larger number of obtained significant hits during the initial homology-screening in BLAST for Bacteria *vs*. Fungi (3583 *vs*. 3168, Fig 1-left) should not be misleading, since this probably represents an artifact of the overrepresentation of bacterially-derived sequences in databases. Among the Fungi, different *Candida* representatives are, by far, the species with most firmly established connection to the RA in the literature [55,56]. On top of this, our study proposes numerous novel fungal pathogens (Table 1) as candidate RA-triggers, being in accordance with underlying closer phylogenetic relation of Fungi to humans, and also to the previously unrecognized RA-triggering potential of these organisms in the literature. Notably, with the much-needed novel studies, aiming to delineate the complex composition of the human mycobiome, fungal commensals could also be recognized as organisms with substantial RA-triggering capacity.

Although no convincing results were obtained for Viruses, partly due to the fact that the analysis could not be fully completed (missing LALIGN step for autoreactivity confirmation), six predicted T cell epitopes that significantly aligned to the RA Antigens 1, 12 and 29, were still identified across three different viral representatives. Two of these belong to the *Marseillevirus* group, which encompasses large dsDNA viruses, characteristic of having a set of core-viral genes complemented with a subset of eukaryotic-host-like genes and eukaryotic-like intergenic sequences, probably inherited *via* horizontal gene transfer, opening an avenue for molecular mimicry due to the possible peptide resemblance [57,58]. Although identified *Marseillevirus* primarily infect amoeba, these can also contact the human host [59,60]. Aside from these viruses, with no connection to the RA across the literature, it should be noted that the initial BLASTp search retrieved a number of viral *PubMed* organisms, like *Influenza* virus and *Mimivirus*, which however did not meet the restrictive filtering criteria applied for obtaining the high-confidence set of predictions, given that the trade-off between true positive and false positive discovery rate cannot straightforwardly be established for analysis of this kind.

Beyond restrictive BLASTp filtering criteria, the high-confidence list of candidate RA-triggering organisms was streamlined further through TepiTool predictions of novel T cell epitopes that get efficiently processed and bound to the RA-related HLA alleles. This assumed two complementary approaches for confirming RA-triggering capacity for blasted organisms: *i*) the retrieval of significant BLASTp hits corresponding to the initial pool of RA-related experimental T-epitopes; *ii*) autoreactivity confirmation through LALIGN for high-affinity (top 0.5%) T-epitopes, against all the RA-related autoantigens. Obtained results revealed high-level bias of predicted top T cell epitopes towards evolutionary conserved Antigen 1, comprising also more than 2/3 of all the significant BLASTp hits. Interestingly, Antigen 1 specific T-cell epitopes fall within a dozen of highly conserved clusters, whose consensus sequences also significantly align to the most conserved portion of the Antigen 1 sequence. The obvious supremacy of this antigen, which corresponds to the Endoplasmic reticulum chaperone BiP from the highly conserved protein family HSP70, can certainly be attributed to its high-level evolutionary conservation and the corresponding distribution throughout all domains of life. However, the fact that Antigen 9 is also designated as the Hsp, just like Antigen 14 and Antigen 30, although much weaker or no impact of these proteins was identified throughout our analysis, seems puzzling. For Antigen 9, homologous sequences among novel T cell epitopes are found, though in smaller quantities, while Antigens 14 and 30 actually correspond to the Hsp-homologs, where the former is deposited in the truncated (fragment) form.

Hsps primarily respond to cellular stress, by alleviating the damage of protein denaturation *via* assisting protein refolding into native 3D conformation, thus also called molecular chaperones. Consistently, these proteins are highly upregulated during inflammation, but also highly immunogenic and efficiently presented within MHC class II molecules to be recognized, among others, by the T regulatory cells too. In numerous autoimmune pathologies, just like in RA, Hsps are recognized as very potent autoantigens [61–63]. Besides these proinflammatory effects, secretory forms of Hsps display important immunomodulatory roles that contribute to the re-establishing of autotolerance *via* IL-10-producing T regulatory cells [64,65], which places this class of proteins into the focus for therapies currently under development, aiming at inducing self-tolerance against most potent autoantigens to achieve long-lasting disease remission with no additional medication needed [66,67]. In this context, the possibility of identifying such autoantigens, provided partly by our analysis also, as evidenced on the example of extensive T epitope characterization for Antigen 1, stands as an important results, since in most autoimmune diseases such autoantigens are still insufficiently characterized.

In summary, results of the presented analysis revealed a wide spectrum of microbial RA-triggering candidates, which are able to elicit RA-specific autoreactive T cells. However, it is important to note that for obtaining a high-confidence set of predictions; restrictive filtering parameters had been applied, which therefore certainly failed to predict a share of RA-triggering organisms. Namely, the 80% sequence identity cut-off, which does not necessarily correlate to the BLASTp alignment E-value, is sufficiently rigorous, as it allows only up to 3 mismatches in the blasted 15mer epitope. This is sufficient enough for triggering TCR cross-reactivity, since not all amino acid residues from the epitope make sequence-specific contacts to the TCR. Moreover, another filtering criterion—no gaps in the alignment allowed—captures the nature of the peptide presentation by MHC molecules, where the geometry of interaction with peptides within certain pockets from the peptide binding cleft should be preserved. Additionally, the starting point in the presented analysis was the current set of RA-related T cell epitopes and autoantigens, which cannot be considered as fully characterized. Besides noted limitations, our study reports a much larger repertoire of putative microbial RA-triggers, than previously acknowledged, providing interesting insights into the underestimated role of Fungi in autoimmunity and the possibility of a more direct route for bacterial commensals to induce RA-pathology. Also, the possibility of identifying most potent, i.e. most likely mimicked autoantigens through our analysis, represents result of a high importance, applicable in a variety of different autoimmune pathologies.

## Supporting information

S1 Table The list of experimental T cell epitopes related to rheumatoid arthritis downloaded from the IEDB database (03.12.2020). (DOCX)

S2 Table List of antigen associated with corresponding unique epitopes. (DOCX)

S3 Table The overall distribution of epitopes BLASTp hits across Bacteria/Fungi/Viruses in terms of originating antigens. (DOCX)

S4 Table The distribution of BLASTp hits across bacterial human pathogen/commensals where the relation between rheumatoid arthritis and the corresponding species has not been previously established in literature (PubMed). (DOCX)

S5 Table The distribution of BLASTp Hits across bacterial human pathogen/commensals where the relation between rheumatoid arthritis and the corresponding species has been previously established in literature (PubMed). (DOCX)

S6 Table The distribution of BLASTp hits across fungi human pathogen/commensals where the relation between rheumatoid arthritis and the corresponding species has not been previously established in literature (PubMed). (DOCX)

S7 Table The distribution of BLASTp hits across fungi human pathogen/commensals where the relation between rheumatoid arthritis and the corresponding species has been previously established in literature (PubMed). (DOCX)

S8 Table The distribution of BLASTp hits across viruses human pathogen/commensals where the relation between rheumatoid arthritis and the corresponding species has not been previously established in literature (PubMed). (DOCX)

S9 Table HLA alleles associated with rheumatoid arthritis. (DOCX)

S10 Table A comprehensive list (homologues to the IEDB experiential and newly predicted epitopes) rheumatoid arthritis triggering epitopes homologous to Antigen_1 (endoplasmic reticulum chaperone BiP). (DOCX)

S1 File (XLSX)

S2 File (XLSX)

S3 File (XLSX)

S4 File (XLSX)

S5 File (XLSX)

S6 File (XLSX)
