# Supplementary material for: Mining the capacity of human-associated microorganisms to trigger rheumatoid arthritis—A systematic immunoinformatics analysis of T cell epitopes
Source: PLoS One. 2021 Jun 29;16(6):e0253918. doi: 10.1371/journal.pone.0253918 (PMC8241107; doi:10.1371/journal.pone.0253918)
Supplement: S1 Table — (DOCX) [file pone.0253918.s001.docx]

Mining the capacity of human-associated microorganisms to trigger rheumatoid arthritis – a systematic immunoinformatics analysis of T cell epitopes

Jelena Repac^1^, Marija Mandić^1^, Tanja Lunić^1^, Bojan Božić^1*¶^, Biljana Božić Nedeljković^1*¶^

^1^ Institute of Physiology and Biochemistry “Ivan Djaja”, Faculty of Biology, University of Belgrade, Belgrade, Serbia

# **S1 Table**. **The list of experimental T cell Epitopes related to Rheumatoid Arthritis downloaded from the IEDB data base (03.12.2020.).**

| No. | Epitope ID | Sequence | Epitope Modified Residue(s) | Epitope Modification |
| --- | --- | --- | --- | --- |
| 1 | 1726 | AGWLADRSVRYPI |  |  |
| 2 | 13572 | ENPVVHFFKNIVTPR |  |  |
| 3 | 16626 | FLGMESCGI |  |  |
| 4 | 19924 | GGMGGGLGGG |  |  |
| 5 | 19925 | GGMGGGMGGG |  |  |
| 6 | 30200 | KDLLEQKRAAVDTYC |  |  |
| 7 | 37700 | LLQDSVDFSL |  |  |
| 8 | 51342 | QLFNHTMFI |  |  |
| 9 | 59411 | SLQEEIAFL |  |  |
| 10 | 69649 | VLMIKALEL |  |  |
| 11 | 106319 | CVDTRSGNCYLDIRP |  |  |
| 12 | 106396 | EPVSGSFTTALDGPS |  |  |
| 13 | 106414 | FFYFFTGSSQLEFDP |  |  |
| 14 | 106470 | GPKGQTGEPGIAGFKGEQGPK |  |  |
| 15 | 106489 | GVVSHSFPATLETQE |  |  |
| 16 | 106500 | HAYSVTGAEEVESNG |  |  |
| 17 | 106546 | ISGLPSGGDDLETST |  |  |
| 18 | 106625 | LSGLPSGGEVLEISV |  |  |
| 19 | 106790 | RSFTLASSETGVG |  |  |
| 20 | 106809 | SAFWPSLPSGLDAAY |  |  |
| 21 | 106964 | VKEGHSPPDDVDIVI |  |  |
| 22 | 107076 | AGFKGEQGPKGEP | HYL(P13) | Hydroxylation |
| 23 | 107222 | PTFGRSFTLASSE |  |  |
| 24 | 107241 | SQRFSKIASNTQSR |  |  |
| 25 | 107248 | TGEPGIAGFKGEQGPKGEPG | HYL(P4, P19) | Hydroxylation |
| 26 | 107265 | VGYDDQESVKSKV |  |  |
| 27 | 107290 | AGMDMCSAGWLADRSVRY |  |  |
| 28 | 107398 | GKPGIAGFKGEQGPKG |  |  |
| 29 | 107402 | GNQWVGYDDQESVKSK |  |  |
| 30 | 107430 | GRSFTLASSETGVGAP |  |  |
| 31 | 107432 | GTSLTIPCYFIDPMH |  |  |
| 32 | 107525 | MGIPTFGRSFTLASSE |  |  |
| 33 | 107530 | NFGSQRFSKIASNTQS |  |  |
| 34 | 107577 | QHLDFISIMTYDFHGA |  |  |
| 35 | 107586 | QWVGYDDQESVKSKVQ |  |  |
| 36 | 107611 | SCFPDALDRFLCTHII |  |  |
| 37 | 107678 | VVLLVATEGRVRVNSAYQ |  |  |
| 38 | 107681 | VVVKGIVFHYRAISTRYT |  |  |
| 39 | 107697 | YLAWQAGMDMCSAGW |  |  |
| 40 | 107730 | AGWLADGSLRYPI |  |  |
| 41 | 107731 | AGWLADQTVRYPI |  |  |
| 42 | 107751 | ATLEVQSLRSNDSGVYRC |  |  |
| 43 | 107857 | GGVGPIGPPGERGA |  |  |
| 44 | 108000 | LGLRLIYFSYDVKMKEKGDI |  |  |
| 45 | 108008 | LLRGYHQDAY |  |  |
| 46 | 108060 | PEFSGLPSGIAEV |  |  |
| 47 | 108096 | RGFTGLQGLPGPPGPSGD |  |  |
| 48 | 108106 | RVAEQLRAYLEGECV |  |  |
| 49 | 108121 | SGFQGLPGPPGPPGEGGGK |  |  |
| 50 | 108138 | SMNVEVTDKGDVPEGYK |  |  |
| 51 | 108142 | SQIVNDFQKGDIAEGYS |  |  |
| 52 | 108208 | WDRETQICKAKAQ |  |  |
| 53 | 108263 | AGFKGEQGPKGEPGP |  |  |
| 54 | 108383 | GIAGFKGEQGPKGEP | GLYC(6K, 12K) | Glycosylation |
| 55 | 108394 | GPEGAQGPRGEPGTP |  |  |
| 56 | 109216 | GPTGARGPEGAQGPR |  |  |
| 57 | 109247 | GSAGAPGIAGAPGFP |  |  |
| 58 | 110595 | GIAGAPGFPGPRGPP |  |  |
| 59 | 110599 | GIPGAKGSAGAPGIA |  |  |
| 60 | 110623 | GRRITTRRIMENGQE |  |  |
| 61 | 110681 | PGMVQQIQSVCMECQ |  |  |
| 62 | 110705 | SGPFFTFSSSFPGHS |  |  |
| 63 | 111357 | GNPGTDGIPGAKGSA |  |  |
| 64 | 111358 | GPAGASGNPGTDGIP |  |  |
| 65 | 111363 | GPRGEPGTPGSPGPA |  |  |
| 66 | 111383 | GTPGSPGPAGASGNP |  |  |
| 67 | 113744 | QDFTNRINKLKNS | CITR(R6) | Citrullination |
| 68 | 114735 | ALSTLVLNRLKVGLQ |  |  |
| 69 | 114753 | AYVLLSEKKISSIQS |  |  |
| 70 | 114829 | GEALSTLVLNRLKVG |  |  |
| 71 | 114868 | KCEFQDAYVLLSEKK |  |  |
| 72 | 114968 | VASLLTTAEVVVTEI |  |  |
| 73 | 117360 | RIHMVYSKRSGKPRGYAFIEY |  |  |
| 74 | 117395 | VEPKVKSKKREAVAGRGRGRGRGRGRGRGRGRGGPRR |  |  |
| 75 | 121030 | VEIRAEGNSRFTY |  |  |
| 76 | 125486 | LPNFSSLNLRETNLDSLPL | CITR(R10) | Citrullination |
| 77 | 125487 | LPNFSSLNLRETNLDSLPL |  |  |
| 78 | 125563 | SSRSYVTTSTRTYSLGSAL | CITR(R3, R11) | Citrullination |
| 79 | 130458 | CSAGWLADRSVRYPISKA |  |  |
| 80 | 130514 | IPCYFIDPMHPVTTAP |  |  |
| 81 | 130522 | KEKEVVLLVATEGRVR |  |  |
| 82 | 130532 | LADRSVRYPISKARPNCG |  |  |
| 83 | 130546 | LQKRSSRHPRRSRPST |  |  |
| 84 | 130582 | QAGMDMCSAGWLADRSVR |  |  |
| 85 | 130587 | QPSPLRVLLGTSLTIP |  |  |
| 86 | 130602 | RVLLGTSLTIPCYFID |  |  |
| 87 | 130603 | RVNSAYQDKVSLPNYP |  |  |
| 88 | 130624 | TTGHVYLAWQAGMDMCSA |  |  |
| 89 | 130626 | VATEGRVRVNSAYQDKV |  |  |
| 90 | 132790 | DILEEERAV |  |  |
| 91 | 132806 | EYWNSQKDL |  |  |
| 92 | 132909 | TELGRPSAEYL |  |  |
| 93 | 133947 | VVLLVATEGRVRVNSAYQDK | CITR(R10) | Citrullination |
| 94 | 133948 | VVLLVATEGRVRVNSAYQDK |  |  |
| 95 | 136219 | FEEYGKIDTIEIIT |  |  |
| 96 | 136590 | RDYFEEYGKIDTIEIIT |  |  |
| 97 | 136731 | VRVFQATRGKLSSKCSVVLG |  |  |
| 98 | 138236 | SAVRARSSVPGVR |  |  |
| 99 | 139481 | DKKREEAPSLRPAPP | CITR(R4, R11) | Citrullination |
| 100 | 139504 | GDKELRTGKEKVTSG | CITR(R6) | Citrullination |
| 101 | 139522 | IEVLKRKVIEKVQHI | CITR(R6) | Citrullination |
| 102 | 139535 | LENLRSKIQKLESDV | CITR(R5) | Citrullination |
| 103 | 139543 | LLQQERPIRNSVDEL | CITR(R6, R9) | Citrullination |
| 104 | 139560 | NRCHAANPNGRYYWG | CITR(R2, R11) | Citrullination |
| 105 | 139569 | PRKQCSKEDGGGWWY | CITR(R2) | Citrullination |
| 106 | 139571 | QKLESDVSAQMEYCR | CITR(R15) | Citrullination |
| 107 | 139585 | QNPGSPRPGSTGTWN | CITR(R7) | Citrullination |
| 108 | 139598 | RPAPPPISGGGYRAR | CITR(R1, R13, R15) | Citrullination |
| 109 | 139604 | SGCRMKGLIDEVNQD | CITR(R4) | Citrullination |
| 110 | 139626 | TTRRSCSKTVTKTVI | CITR(R3, R4) | Citrullination |
| 111 | 139631 | VIQNRQDGSVDFGRK | CITR(R5, R14) | Citrullination |
| 112 | 139640 | WYNRCHAANPNGRYY | CITR(R4, R13) | Citrullination |
| 113 | 139641 | WYSMRKMSMKIRPFF | CITR(R5, R12) | Citrullination |
| 114 | 141284 | GGGYRARPAKAAATQ | CITR(R7) | Citrullination |
| 115 | 141289 | GKLYGIRDVRSTRDR | CITR(R7, R10) | Citrullination |
| 116 | 141474 | YAEYHFRVGSEAEGY | CITR(R7) | Citrullination |
| 117 | 153365 | CDMNTENGGWTVIQN |  |  |
| 118 | 153374 | DVSAQMEYCRTPCTV |  |  |
| 119 | 153375 | DWPFCSDEDWNYKCP |  |  |
| 120 | 153376 | EAVQSTSSSSQFYMY |  |  |
| 121 | 153384 | FTNRINKLKNSLFEY |  |  |
| 122 | 153385 | GDKELRTGKEKVTSG |  |  |
| 123 | 153394 | GSSERGSAGHWTSES |  |  |
| 124 | 153401 | IRVLRSILENLRSKI | CITR(R2, R5, R12) | Citrullination |
| 125 | 153404 | ITSDPRKQCSKEDGG | CITR(R6) | Citrullination |
| 126 | 153408 | LENLRSKIQKLESDV |  |  |
| 127 | 153412 | LLQQERPIRNSVDEL |  |  |
| 128 | 153413 | LMGENRTMTIHNGMF |  |  |
| 129 | 153433 | NSNIPTNLRVLRSIL |  |  |
| 130 | 153439 | PRKQCSKEDGGGWWY |  |  |
| 131 | 153442 | QKLESDVSAQMEYCR |  |  |
| 132 | 153443 | QKQLEQVIAKDLLPS |  |  |
| 133 | 153444 | QMEYCRTPCTVSCNI |  |  |
| 134 | 153446 | QNPGSPRPGSTGTWN |  |  |
| 135 | 153454 | RPAPPPISGGGYRAR |  |  |
| 136 | 153459 | SETESRGSESGIFTN |  |  |
| 137 | 153461 | SGCRMKGLIDEVNQD |  |  |
| 138 | 153462 | SGPGSTGNRNPGSSG |  |  |
| 139 | 153478 | TPCTVSCNIPVVSGK |  |  |
| 140 | 153484 | TTRRSCSKTVTKTVI |  |  |
| 141 | 153485 | TVQNEANKYQISVNK |  |  |
| 142 | 153486 | VDIDIKIRSCRGSCS | CITR(R8, R11) | Citrullination |
| 143 | 153487 | VDIDIKIRSCRGSCS |  |  |
| 144 | 153495 | VSPGTRREYHTEKLV |  |  |
| 145 | 153496 | VVWMNWKGSWYSMRK |  |  |
| 146 | 153501 | WYNRCHAANPNGRYY |  |  |
| 147 | 153502 | WYSMRKMSMKIRPFF |  |  |
| 148 | 153503 | YCGLPGEYWLGNDKI |  |  |
| 149 | 156882 | GVYATRSSAVRLR |  |  |
| 150 | 156883 | GVYATRSSAVRLRSSVPGVR | CITR(R6, R11, R13) | Citrullination |
| 151 | 156884 | GVYATRSSAVRLRSSVPGVR |  |  |
| 152 | 167212 | APEEISAMVLTKMKETAEAY |  |  |
| 153 | 167215 | ATNGDTHLGGEDFDQRVMEH |  |  |
| 154 | 167222 | DAGTIAGLNVMRIINEPTAA |  |  |
| 155 | 167228 | DIKFLPFKVVEKKTKPYIQV |  |  |
| 156 | 167230 | DNQPTVTIKVYEGERPLTKD |  |  |
| 157 | 167233 | DVSLLTIDNGVFEVVATNGD |  |  |
| 158 | 167235 | EDKKEDVGTVVGIDLGTTYS |  |  |
| 159 | 167238 | EIIANDQGNRITPSYVAFTP |  |  |
| 160 | 167239 | EKFAEEDKKLKERIDTRNEL |  |  |
| 161 | 167242 | EPTAAAIAYGLDKREGEKNI |  |  |
| 162 | 167245 | ESHQDADIEDFKAKKKELEE |  |  |
| 163 | 167250 | EVEKAKRALSSQHQARIEIE |  |  |
| 164 | 167254 | FEIDVNGILRVTAEDKGTGN |  |  |
| 165 | 167264 | GEKNILVFDLGGGTFDVSLL |  |  |
| 166 | 167266 | GIPPAPRGVPQIEVTFEIDV |  |  |
| 167 | 167267 | GKEPSRGINPDEAVAYGAAV |  |  |
| 168 | 167272 | GSAGPPPTGEEDTAELHHHH |  |  |
| 169 | 167273 | GTTYSCVGVFKNGRVEIIAN |  |  |
| 170 | 167283 | IETVGGVMTKLIPRNTVVPT |  |  |
| 171 | 167285 | IGDKEKLGGKLSSEDKETME |  |  |
| 172 | 167295 | KDVRKDNRAVQKLRREVEKA |  |  |
| 173 | 167296 | KELEEIVQPIISKLYGSAGP |  |  |
| 174 | 167297 | KETMEKAVEEKIEWLESHQD |  |  |
| 175 | 167298 | KGTGNKNKITITNDQNRLTP |  |  |
| 176 | 167302 | KNQLTSNPENTVFDAKRLIG |  |  |
| 177 | 167303 | KRLIGRTWNDPSVQQDIKFL |  |  |
| 178 | 167318 | LKKSDIDEIVLVGGSTRIPK |  |  |
| 179 | 167348 | NRLTPEEIERMVNDAEKFAE |  |  |
| 180 | 167359 | PLTKDNHLLGTFDLTGIPPA |  |  |
| 181 | 167364 | PYIQVDIGGGQTKTFAPEEI |  |  |
| 182 | 167387 | RIEIESFYEGEDFSETLTRA |  |  |
| 183 | 167396 | RSTMKPVQKVLEDSDLKKSD |  |  |
| 184 | 167397 | RVMEHFIKLYKKKTGKDVRK |  |  |
| 185 | 167413 | TAEAYLGKKVTHAVVTVPAY |  |  |
| 186 | 167418 | TGDLVLLDVCPLTLGIETVG |  |  |
| 187 | 167423 | TLTRAKFEELNMDLFRSTMK |  |  |
| 188 | 167426 | TRIPKIQQLVKEFFNGKEPS |  |  |
| 189 | 167427 | TRNELESYAYSLKNQIGDKE |  |  |
| 190 | 167434 | TVPAYFNDAQRQATKDAGTI |  |  |
| 191 | 167436 | TVVPTKKSQIFSTASDNQPT |  |  |
| 192 | 167438 | VAFTPEGERLIGDAAKNQLT |  |  |
| 193 | 167461 | YGAAVQAGVLSGDQDTGDLV |  |  |
| 194 | 180071 | QYMRADQAAGGLR | CITR(R4) | Citrullination |
| 195 | 180072 | SAVRARSSVPGVR | CITR(R6) | Citrullination |
| 196 | 188640 | AGWLARRSVRYPI | CITR(R6) | Citrullination |
| 197 | 188641 | AGWLARRSVRYPI |  |  |
| 198 | 222599 | GVYATRSSAVRLR | CITR(R6) | Citrullination |
| 199 | 227746 | ATIKAEFVRAETPYM | CITR(R9) | Citrullination |
| 200 | 227773 | GYRARPAKAAAT | CITR(R5, K8) | Citrullination |
| 201 | 227780 | IFDSRGNPTVEVDLF | CITR(R5) | Citrullination |
| 202 | 227781 | IFDSRGNPTVEVDLF |  |  |
| 203 | 227793 | KRIAKAVNEKSCNCL | CITR(R2) | Citrullination |
| 204 | 227794 | KRIAKAVNEKSCNCL |  |  |
| 205 | 227848 | SSLNLRETNLDSL | CITR(R6) | Citrullination |
| 206 | 422040 | LTQRGSVLR | CITR(R4) | Citrullination |
| 207 | 422106 | VETRDGQVI | CITR(R4) | Citrullination |
| 208 | 423045 | QLFNKHTMFI |  |  |
| 209 | 536386 | KAKFAGRNFRNPLAK |  |  |
| 210 | 537027 | MSILKIHAREIFDSR |  |  |
| 211 | 538244 | VIGMDVAASEFFRSG |  |  |
| 212 | 538627 | APGNRGFPGQDGLAG | CITR(R5) | Citrullination |
| 213 | 549063 | AGFKGEQGKGEP | MCM(K4) | Main chain modification |
| 214 | 549064 | AGFKGEQGKGEP |  |  |
| 215 | 549604 | MDMCSAGWLADRSVR | CITR(R12) | Citrullination |
| 216 | 566588 | KAKFAGRNFRNPLAK | CITR(R7, R10) | Citrullination |
| 217 | 566598 | LPVGAANFREAMRIG | CITR(R9, R13) | Citrullination |
| 218 | 566599 | LPVGAANFREAMRIG |  |  |
| 219 | 566602 | MSILKIHAREIFDSR | CITR(R9, R15) | Citrullination |
| 220 | 566626 | RYMGKGVSKAVEHIN | CITR(R1) | Citrullination |
| 221 | 566627 | RYMGKGVSKAVEHIN |  |  |
| 222 | 566637 | TSKGLFRAAVPSGAS | CITR(R7) | Citrullination |
| 223 | 566638 | TSKGLFRAAVPSGAS |  |  |
| 224 | 566640 | VIGMDVAASEFFRSG | CITR(R13) | Citrullination |
| 225 | 886761 | DAGWLADQTVRYPIHT | CITR(R11) | Citrullination |
| 226 | 886765 | DEFPGVRTYGIRDTNETYDV | CITR(R7, R12) | Citrullination |
| 227 | 886791 | DVYCFVDRLEGEVFFA | CITR(R8) | Citrullination |
| 228 | 886873 | GYEQCDAGWLRDQTVRYPIV | CITR(R11, R16) | Citrullination |
| 229 | 886920 | KCYAGWLADGSLRYPIV | CITR(R13) | Citrullination |
| 230 | 887042 | PGVRTYGVRPSTETYDVY | CITR(R4, R9) | Citrullination |
| 231 | 887163 | STRYTLDFDRAQRACLQ | CITR(R3, R1, R13) | Citrullination |
| 232 | 1069340 | DPGVEVTLTMKVASGSTGDQ |  |  |
| 233 | 1309419 | AGFKGEQGPKGEPGPAGP | GLYC(K10) | Glycosylation |
| 234 | 1309420 | AGFKGEQGPKGEPGPAGP | GLYC(K4) | Glycosylation |
