# Supplementary material for: Mining the capacity of human-associated microorganisms to trigger rheumatoid arthritis—A systematic immunoinformatics analysis of T cell epitopes
Source: PLoS One. 2021 Jun 29;16(6):e0253918. doi: 10.1371/journal.pone.0253918 (PMC8241107; doi:10.1371/journal.pone.0253918)
Supplement: S2 Table — (DOCX) [file pone.0253918.s002.docx]

Mining the capacity of human-associated microorganisms to trigger rheumatoid arthritis – a systematic immunoinformatics analysis of T cell epitopes

Jelena Repac^1^, Marija Mandić^1^, Tanja Lunić^1^, Bojan Božić^1*¶^, Biljana Božić Nedeljković^1*¶^

^1^ Institute of Physiology and Biochemistry “Ivan Djaja”, Faculty of Biology, University of Belgrade, Belgrade, Serbia

# **S2 Table.** **List of antigen associated with corresponding unique epitopes.**

| No. | Accession | Antigen Name | Epitope Number^a^ |
| --- | --- | --- | --- |
| 1 | P11021 | Endoplasmic reticulum chaperone BiP | 152–193 |
| 2 | P02675 | Fibrinogen beta chain | 99, 102–106, 108, 111–114, 117, 118, 120, 124, 125, 128, 129, 133, 139, 141, 145, 148, 200 |
| 3 | P02671 | Fibrinogen alpha chain | 67, 100, 101, 107, 109, 110, 116, 119, 121, 123, 132, 136, 138, 142, 144, 206 |
| 4 | P16112 | Aggrecan core protein | 1, 18, 27, 37, 38, 40–42, 46, 93, 196, 225–231 |
| 5 | P06733 | Alpha-enolase | 201, 203, 209–211, 217, 220, 222 |
| 6 | A0A087X1T7 | Aggrecan core protein | 31, 39, 79–89, 215 |
| 7 | P36222 | Chitinase-3-like protein 1 | 19, 23, 24, 26, 29, 30, 32–36 |
| 8 | P05783 | Keratin type I cytoskeletal 18 | 7, 9, 76, 78, 149, 150, 205, 207 |
| 9 | P10809 | 60 kDa heat shock protein mitochondrial | 5, 68–72 |
| 10 | P02458 | Collagen alpha-1(II) chain | 14, 22, 25, 43, 47, 49, 53–59, 63–66, 75, 194, 212, 213, 233 |
| 11 | Q2L6G2 | HLA class I histocompatibility antigen B alpha chain | 45, 48, 52 |
| 12 | P35579 | Myosin-9 | 8, 10, 208 |
| 13 | P08254 | Stromelysin-1 | 13, 20 |
| 14 | C9JRD2 | DnaJ homolog subfamily B member 2 (Fragment) | 60, 62 |
| 15 | P22626 | Heterogeneous nuclear ribonucleoproteins A2/B1 | 95, 96 |
| 16 | O75339 | Cartilage intermediate layer protein 1 | 115, 199 |
| 17 | P08670 | Vimentin | 98 |
| 18 | Q9UM07 | Protein-arginine deiminase type-4 | 97, 232 |
| 19 | J3QL64 | Myelin basic protein | 2 |
| 20 | P60709 | Actin cytoplasmic 1 | 3 |
| 21 | P35555 | Fibrillin-1 | 11 |
| 22 | P24821 | Tenascin | 12 |
| 23 | P03956 | Interstitial collagenase | 15 |
| 24 | P17655 | Calpain-2 catalytic subunit | 16 |
| 25 | Q6UVK1 | Chondroitin sulfate proteoglycan 4 | 17 |
| 26 | P51512 | Matrix metalloproteinase-16 | 21 |
| 27 | A0A5B6 | T cell receptor beta variable 28 | 44 |
| 28 | A0A087WT01 | T cell receptor beta variable 27 | 50 |
| 29 | A0A075B6N1 | T cell receptor beta variable 19 | 51 |
| 30 | P31689 | DnaJ homolog subfamily A member 1 | 61 |
| 31 | P08621 | U1 small nuclear ribonucleoprotein 70 kDa | 73 |
| 32 | P62314 | Small nuclear ribonucleoprotein Sm D1 | 74 |
| 33 | P02538 | Keratin, type II cytoskeletal 6A | 4 |
| 34 | A2BFX2 | MHC HLA-DR4-beta chain | 6 |
| 35 | Q5EP54 | MHC class II antigen | 90 |
| 36 | P01911 | HLA-DRB1 beta | 91 |
| 37 | SRC241953^*^ (AAK31610.1) | MHC class II antigen | 92 |

^a^ Epitope number as assigned in S1 Table.

^*^ Accession number provided in IEDB was invalid. The corresponding protein sequence (AAK31610.1) was retrieved from the reference provided therein [[1](#_ENREF_1)].

# **SI_References**

1. Massa M, Mazzoli F, Pignatti P, De Benedetti F, Passalia M, Viola S, et al. Proinflammatory responses to self HLA epitopes are triggered by molecular mimicry to Epstein‐Barr virus proteins in oligoarticular juvenile idiopathic arthritis. Arthritis Rheum. 2002;46(10):2721-9.
