# Supplementary material for: Mining the capacity of human-associated microorganisms to trigger rheumatoid arthritis—A systematic immunoinformatics analysis of T cell epitopes
Source: PLoS One. 2021 Jun 29;16(6):e0253918. doi: 10.1371/journal.pone.0253918 (PMC8241107; doi:10.1371/journal.pone.0253918)
Supplement: S3 Table — (DOCX) [file pone.0253918.s003.docx]

Mining the capacity of human-associated microorganisms to trigger rheumatoid arthritis – a systematic immunoinformatics analysis of T cell epitopes

Jelena Repac^1^, Marija Mandić^1^, Tanja Lunić^1^, Bojan Božić^1*¶^, Biljana Božić Nedeljković^1*¶^

^1^ Institute of Physiology and Biochemistry “Ivan Djaja”, Faculty of Biology, University of Belgrade, Belgrade, Serbia

# **S3 Table. The overall distribution of Epitopes BLASTp Hits across Bacteria/Fungi/Viruses in terms of originating antigens.**

| BACTERIA/FUNGI/VIRUSES | | | |
| --- | --- | --- | --- |
| Ag No. | **Number of  Unique Epitopes** | **Number of BLASTp hits** | **Number of Unique BLASTp hits** |
| 1 | 42 | 1999/2482/551 | 1558/1208/151 |
| 2 | 24 | 92/12/1 | 33/10/1 |
| 3 | 16 | 6/4/3 | 6/4/3 |
| 4 | 18 | 43/5/0 | 37/5/0 |
| 5 | 8 | 345/353/4 | 327/265/4 |
| 6 | 14 | 6/6/0 | 6/6/0 |
| 7 | 11 | 100/42/0 | 99/37/0 |
| 8 | 8 | 5/1/0 | 4/1/0 |
| 9 | 6 | 208/155/25 | 204/100/15 |
| 10 | 22 | 710/88/186 | 433/78/115 |
| 11 | 3 | 32/1/0 | 32/1/0 |
| 12 | 3 | 2/2/0 | 2/2/0 |
| 13 | 2 | 0/0/1 | 0/0/1 |
| 14 | 2 | 7/0/0 | 7/0/0 |
| 15 | 2 | 1/0/0 | 1/0/0 |
| 16 | 2 | 2/0/0 | 2/0/0 |
| 17 | 1 | 1/0/100 | 1/0/100 |
| 18 | 2 | 0/0/0 | 0/0/0 |
| 19 | 1 | 3/0/0 | 3/0/0 |
| 20 | 1 | 0/1/0 | 0/1/0 |
| 21 | 1 | 0/1/ | 0/1/0 |
| 22 | 1 | 1/0/0 | 1/0/0 |
| 23 | 1 | 0/0/0 | 0/0/0 |
| 24 | 1 | 0/0/0 | 0/0/0 |
| 25 | 1 | 0/0/0 | 0/0/0 |
| 26 | 1 | 0/1/0 | 0/1/0 |
| 27 | 1 | 0/0/0 | 0/0/0 |
| 28 | 1 | 0/0/0 | 0/0/0 |
| 29 | 1 | 1/0/0 | 1/0/0 |
| 30 | 1 | 0/0/0 | 0/0/0 |
| 31 | 1 | 0/0/0 | 0/0/0 |
| 32 | 1 | 0/0/0 | 0/0/0 |
| 33 | 1 | 0/14/5 | 0/14/3 |
| 34 | 1 | 13/0/0 | 13/0/3 |
| 35 | 1 | 0/0/0 | 0/0/3 |
| 36 | 1 | 5/0/1 | 5/0/1 |
| 37 | 1 | 0/0/1 | 0/0/1 |
