# Supplementary material for: Mining the capacity of human-associated microorganisms to trigger rheumatoid arthritis—A systematic immunoinformatics analysis of T cell epitopes
Source: PLoS One. 2021 Jun 29;16(6):e0253918. doi: 10.1371/journal.pone.0253918 (PMC8241107; doi:10.1371/journal.pone.0253918)
Supplement: S4 Table — (DOCX) [file pone.0253918.s004.docx]

Mining the capacity of human-associated microorganisms to trigger rheumatoid arthritis – a systematic immunoinformatics analysis of T cell epitopes

Jelena Repac^1^, Marija Mandić^1^, Tanja Lunić^1^, Bojan Božić^1*¶^, Biljana Božić Nedeljković^1*¶^

^1^ Institute of Physiology and Biochemistry “Ivan Djaja”, Faculty of Biology, University of Belgrade, Belgrade, Serbia

# **S4 Table.** **The distribution of BLASTp hits across Bacterial human pathogen/commensals where the relation between rheumatoid arthritis and the corresponding species has not been previously established in literature (PubMed).**

| Bacteria | | | | |
| --- | --- | --- | --- | --- |
| Accession Number | **Epitope Number** | **Start** | **Stop** | **e value** |
| *Acetobacter indonesiensis* | | | | |
| WP_048844353.1 | 185 | 126 | 145 | 4.07e-07 |
| *Acidipropionibacterium timonense* | | | | |
| WP_130864686.1 | 157 | 177 | 196 | 3.95e-04 |
| *Bdellovibrio bacteriovorus* | | | | |
| WP_061836789.1 | 178 | 321 | 339 | 2.05e-07 |
|  | ***Butyrivibrio crossotus*** |  |  |  |
| WP_005602514.1 | 220 | 60 | 74 | 0.001 |
| MBD9030154.1 | 220 | 60 | 74 | 0.001 |
| OKZ37561.1 | 220 | 60 | 74 | 0.001 |
| *Cellulomonas hominis* | | | | |
| WP_179687920.1 | 179 | 501 | 514 | 0.002 |
|  | ***Gemmata obscuriglobus*** |  |  |  |
| WP_010044127.1 | 158 | 7 | 17 | 0.77 |
| *Hazenella coriacea* | | | | |
| WP_131924810.1 | 158 | 4 | 14 | 0.77 |
| *Laceyella sacchari* | | | | |
| WP_132223112.1 | 158 | 4 | 14 | 0.77 |
| WP_102991277.1 | 158 | 4 | 14 | 0.77 |
| *Lachnoanaerobaculum gingivalis* | | | | |
| WP_128675204.1 | 220 | 60 | 74 | 0.001 |
| *Lachnoanaerobaculum saburreum* | | | | |
| WP_008750951.1 | 220 | 60 | 74 | 0.001 |
| WP_008754769.1 | 220 | 60 | 74 | 0.001 |
| *Lachnoanaerobaculum sp. ICM7* | | | | |
| WP_009662719.1 | 220 | 60 | 74 | 0.001 |
| *Lachnoanaerobaculum umeaense* | | | | |
| WP_111525909.1 | 220 | 60 | 74 | 0.001 |
| *Lactiplantibacillus plantarum* | | | | |
| WP_131068520.1 | 2 | 28 | 42 | 4.43e-05 |
| WP_136139271.1 | 2 | 98 | 109 | 0.011 |
| *Lactonifactor longoviformis* | | | | |
| WP_084067596.1 | 53 | 47 | 58 | 0.95 |
| WP_084067596.1 | 53 | 71 | 82 | 0.95 |
| *Limosilactobacillus fermentum* | | | | |
| WP_193817318.1 | 48 | 60 | 74 | 6.98e-04 |
| *Mageeibacillus indolicus* | | | | |
| WP_012993518.1 | 191 | 387 | 405 | 2.05e-07 |
| WP_034574195.1 | 191 | 387 | 405 | 2.05e-07 |
| *Methylorubrum zatmanii* | | | | |
| WP_192285183.1 | 233 | 165 | 179 | 0.013 |
| *Microbacterium arborescens* | | | | |
| WP_162815211.1 | 68 | 197 | 211 | 0.005 |
| WP_162815211.1 | 70 | 195 | 209 | 0.007 |
| WP_114589619.1 | 152 | 116 | 134 | 4.65e-09 |
| WP_114598916.1 | 152 | 140 | 159 | 9.60e-12 |
| WP_114598928.1 | 152 | 116 | 134 | 4.65e-09 |
| WP_170138648.1 | 152 | 112 | 131 | 7.54e-11 |
| WP_114598916.1 | 153 | 245 | 264 | 4.83e-12 |
| WP_114598916.1 | 154 | 185 | 204 | 1.66e-09 |
| WP_114598916.1 | 156 | 455 | 474 | 1.45e-07 |
| WP_170138648.1 | 156 | 431 | 450 | 1.14e-06 |
| WP_114598916.1 | 157 | 230 | 249 | 1.06e-10 |
| WP_114589619.1 | 159 | 27 | 45 | 4.65e-09 |
| WP_114598916.1 | 159 | 51 | 69 | 1.50e-10 |
| WP_114598928.1 | 159 | 27 | 45 | 4.65e-09 |
| WP_170138648.1 | 159 | 24 | 42 | 4.65e-09 |
| WP_114598916.1 | 161 | 200 | 219 | 9.28e-09 |
| WP_114598916.1 | 163 | 290 | 309 | 2.27e-06 |
| WP_114598916.1 | 164 | 500 | 519 | 4.07e-07 |
| WP_114598916.1 | 165 | 215 | 234 | 1.18e-09 |
| WP_114598916.1 | 167 | 380 | 399 | 2.12e-10 |
| WP_114589619.1 | 169 | 12 | 31 | 2.05e-07 |
| WP_114598916.1 | 169 | 36 | 55 | 2.99e-10 |
| WP_114598928.1 | 169 | 12 | 31 | 2.05e-07 |
| WP_170138648.1 | 169 | 9 | 28 | 1.41e-04 |
| WP_114598916.1 | 170 | 425 | 444 | 1.50e-10 |
| WP_114598916.1 | 172 | 275 | 294 | 2.12e-10 |
| WP_114598916.1 | 175 | 515 | 534 | 4.07e-07 |
| WP_114598916.1 | 176 | 81 | 100 | 2.99e-10 |
| WP_170138648.1 | 176 | 53 | 72 | 5.03e-05 |
| WP_114598916.1 | 179 | 530 | 549 | 1.84e-08 |
| WP_114598916.1 | 180 | 470 | 489 | 9.28e-09 |
| WP_170138648.1 | 180 | 447 | 465 | 1.03e-07 |
| WP_114598916.1 | 182 | 306 | 324 | 1.66e-09 |
| WP_114598916.1 | 184 | 260 | 279 | 5.94e-10 |
| WP_114598916.1 | 185 | 155 | 174 | 1.07e-10 |
| WP_114589619.1 | 187 | 298 | 314 | 9.00e-06 |
| WP_114598916.1 | 187 | 320 | 339 | 7.56e-11 |
| WP_114598928.1 | 187 | 298 | 314 | 9.00e-06 |
| WP_114598916.1 | 188 | 365 | 384 | 7.56e-11 |
| WP_114598916.1 | 189 | 561 | 579 | 4.52e-06 |
| WP_114598916.1 | 191 | 440 | 459 | 3.67e-08 |
| WP_114598916.1 | 192 | 66 | 85 | 2.60e-08 |
| WP_114598916.1 | 193 | 395 | 410 | 7.09e-05 |
| WP_114598922.1 | 201 | 11 | 24 | 4.20e-05 |
| WP_114598922.1 | 202 | 11 | 24 | 4.20e-05 |
| WP_114598922.1 | 211 | 242 | 252 | 0.060 |
| *Microcystis aeruginosa LG13-12* | | | | |
| NCR72816.1 | 166 | 455 | 474 | 1.36e-11 |
| *Microcystis aeruginosa LG13-13* | | | | |
| NCQ92557.1 | 166 | 455 | 474 | 1.36e-11 |
| *Moorea producens* | | | | |
| WP_009149231.1 | 166 | 456 | 475 | 1.36e-11 |
| WP_071106392.1 | 166 | 456 | 475 | 1.36e-11 |
| WP_070395167.1 | 166 | 456 | 475 | 1.36e-11 |
| WP_071103221.1 | 170 | 395 | 414 | 1.45e-07 |
| WP_008186736.1 | 170 | 395 | 414 | 8.10e-07 |
| WP_070391749.1 | 170 | 395 | 414 | 8.10e-07 |
| *Mycobacteroides abscessus subsp. massiliense* | | | | |
| SLB09724.1 | 233 | 82 | 95 | 0.018 |
| SLB58070.1 | 233 | 51 | 64 | 0.018 |
| *Myroides odoratus* | | | | |
| WP_156424224.1 | 159 | 29 | 47 | 4.82e-09 |
| WP_156424224.1 | 169 | 14 | 33 | 2.56e-05 |
| WP_156424224.1 | 176 | 58 | 77 | 5.08e-05 |
| *Negativicoccus succinicivorans* | | | | |
| WP_159821878.1 | 167 | 330 | 349 | 6.38e-06 |
| *Negativicoccus succinicivorans DORA_17_25* | | | | |
| ETI85887.1 | 167 | 330 | 349 | 6.38e-06 |
| *Nodularia spumigena* | | | | |
| WP_063874440.1 | 164 | 471 | 489 | 2.27e-06 |
| *Nosocomiicoccus massiliensis* | | | | |
| WP_040928391.1 | 185 | 100 | 119 | 2.05e-07 |
| *Paraclostridium dentum* | | | | |
| WP_170075967.1 | 104 | 333 | 347 | 8.39e-05 |
| WP_170075967.1 | 105 | 318 | 332 | 2.66e-06 |
| WP_170075967.1 | 111 | 181 | 193 | 0.042 |
| WP_170075967.1 | 112 | 331 | 345 | 2.11e-05 |
| WP_170075967.1 | 125 | 317 | 328 | 0.34 |
| WP_170075967.1 | 128 | 288 | 301 | 0.030 |
| WP_170075968.1 | 142 | 125 | 138 | 0.005 |
| WP_170075967.1 | 145 | 364 | 375 | 1.67e-04 |
| *Parascardovia denticolens* | | | | |
| WP_006289442.1 | 154 | 132 | 151 | 1.66e-09 |
| WP_006291415.1 | 154 | 132 | 151 | 1.66e-09 |
| *Pelomonas puraquae* | | | | |
| WP_088483386.1 | 156 | 432 | 448 | 2.80e-04 |
| *Pelomonas saccharophila* | | | | |
| WP_132575539.1 | 156 | 432 | 448 | 2.80e-04 |
| *Peptidiphaga gingivicola* | | | | |
| WP_064230829.1 | 180 | 430 | 447 | 3.96e-04 |
| *Planktothrix agardhii* | | | | |
| WP_027254455.1 | 178 | 324 | 342 | 1.31e-08 |
| WP_042155265.1 | 178 | 324 | 342 | 1.31e-08 |
| WP_141294475.1 | 178 | 324 | 342 | 1.31e-08 |
| WP_036834349.1 | 178 | 265 | 283 | 1.31e-08 |
| *Prevotella shahii* | | | | |
| MBF1591215.1 | 186 | 382 | 400 | 1.41e-04 |
| MBF1576751.1 | 186 | 382 | 400 | 1.41e-04 |
| MBF1568087.1 | 186 | 382 | 400 | 1.41e-04 |
| *Risungbinella massiliensis* | | | | |
| WP_044641845.1 | 158 | 4 | 14 | 0.77 |
| *Salmonella enterica subsp. enterica serovar Typhi* | | | | |
| NRL40638.1 | 48 | 151 | 165 | 0.060 |
| *Scardovia inopinata* | | | | |
| WP_006293406.1 | 154 | 132 | 151 | 1.66e-09 |
| *Scardovia wiggsiae* | | | | |
| WP_007148200.1 | 154 | 132 | 151 | 1.66e-09 |
| MBF1674605.1 | 154 | 132 | 151 | 1.66e-09 |
| MBF1667389.1 | 154 | 132 | 151 | 1.66e-09 |
| *Sphingobium yanoikuyae* | | | | |
| WP_125999951.1 | 190 | 141 | 160 | 3.81e-11 |
| *Thermoactinomyces vulgaris* | | | | |
| KPC77548.1 | 158 | 4 | 14 | 0.77 |
| *Trueperella pyogenes* | | | | |
| WP_126919898.1 | 49 | 117 | 131 | 0.011 |
| WP_167801386.1 | 49 | 117 | 131 | 0.47 |
