# Supplementary material for: Mining the capacity of human-associated microorganisms to trigger rheumatoid arthritis—A systematic immunoinformatics analysis of T cell epitopes
Source: PLoS One. 2021 Jun 29;16(6):e0253918. doi: 10.1371/journal.pone.0253918 (PMC8241107; doi:10.1371/journal.pone.0253918)
Supplement: S5 Table — (DOCX) [file pone.0253918.s005.docx]

Mining the capacity of human-associated microorganisms to trigger rheumatoid arthritis – a systematic immunoinformatics analysis of T cell epitopes

Jelena Repac^1^, Marija Mandić^1^, Tanja Lunić^1^, Bojan Božić^1*¶^, Biljana Božić Nedeljković^1*¶^

^1^ Institute of Physiology and Biochemistry “Ivan Djaja”, Faculty of Biology, University of Belgrade, Belgrade, Serbia

# **S5 Table. The distribution of BLASTp hits across bacterial human pathogen/commensals where the relation between rheumatoid arthritis and the corresponding species has been previously established in literature (PubMed).**

| Bacteria | | | | | |
| --- | --- | --- | --- | --- | --- |
| Accession Number | **Epitope Number** | | **Start** | **Stop** | **e value** |
| *Bacillus subtilis* | | | | | |
| WP_195850973.1 | | 233 | 82 | 95 | 0.018 |
| *Bacteroides fragilis* | | | | | |
| WP_148661995.1 | | 167 | 124 | 143 | 2.30e-06 |
| WP_148661995.1 | | 188 | 109 | 126 | 5.82e-07 |
| WP_148661995.1 | | 193 | 139 | 152 | 0.14 |
| *Campylobacter jejuni* | | | | | |
| TKH92554.1 | | 48 | 191 | 205 | 6.72e-04 |
| *Capnocytophaga gingivalis* | | | | | |
| ATA86219.1 | | 22 | 144 | 154 | 0.23 |
| WP_002670581.1 | | 22 | 134 | 144 | 0.23 |
| WP_157909410.1 | | 22 | 134 | 144 | 0.23 |
| ATA86219.1 | | 25 | 138 | 154 | 0.098 |
| WP_002670581.1 | | 25 | 128 | 144 | 0.098 |
| WP_157909410.1 | | 25 | 128 | 144 | 0.098 |
| ATA86219.1 | | 53 | 144 | 154 | 0.34 |
| WP_002670581.1 | | 53 | 134 | 144 | 0.34 |
| WP_157909410.1 | | 53 | 134 | 144 | 0.34 |
| ATA86219.1 | | 54 | 144 | 154 | 0.34 |
| WP_002670581.1 | | 54 | 134 | 144 | 0.34 |
| WP_157909410.1 | | 54 | 134 | 144 | 0.34 |
| *Collinsella aerofaciens* | | | | | |
| WP_099431990.1 | | 158 | 7 | 18 | 0.39 |
| WP_195192725.1 | | 158 | 4 | 14 | 0.77 |
| WP_195362288.1 | | 158 | 4 | 14 | 1 |
| *Collinsella sp. An271* | | | | | |
| WP_087201695.1 | | 220 | 58 | 72 | 0.001 |
| *Escherichia coli* | | | | | |
| WP_187269419.1 | | 6 | 42 | 56 | 0.061 |
| TXP94238.1 | | 6 | 67 | 81 | 0.061 |
| WP_187265136.1 | | 48 | 54 | 68 | 2.47e-04 |
| WP_171485496.1 | | 48 | 94 | 108 | 0.005 |
| WP_149785950.1 | | 48 | 92 | 106 | 0.042 |
| TXP33984.1 | | 48 | 78 | 92 | 2.44e-04 |
| 4H1L_H | | 51 | 47 | 63 | 3.22e-08 |
| WP_192967839.1 | | 55 | 113 | 125 | 0.67 |
| WP_192967839.1 | | 55 | 113 | 125 | 0.67 |
| WP_181664680.1 | | 55 | 212 | 226 | 0.48 |
| WP_181663728.1 | | 55 | 364 | 376 | 0.67 |
| WP_181663728.1 | | 55 | 418 | 430 | 0.67 |
| WP_180494555.1 | | 55 | 148 | 160 | 0.67 |
| WP_180494555.1 | | 55 | 220 | 232 | 0.67 |
| WP_180494555.1 | | 55 | 292 | 304 | 0.67 |
| WP_180494555.1 | | 55 | 364 | 376 | 0.67 |
| WP_180494555.1 | | 55 | 454 | 466 | 0.67 |
| WP_180494555.1 | | 55 | 580 | 592 | 0.67 |
| WP_180494500.1 | | 55 | 256 | 268 | 0.67 |
| WP_180494500.1 | | 55 | 310 | 322 | 0.67 |
| WP_180494500.1 | | 55 | 238 | 250 | 0.95 |
| WP_173671570.1 | | 55 | 226 | 238 | 0.67 |
| WP_173671570.1 | | 55 | 262 | 274 | 0.67 |
| WP_173671570.1 | | 55 | 280 | 292 | 0.67 |
| WP_143370530.1 | | 55 | 238 | 250 | 0.67 |
| SQP82655.1 | | 55 | 472 | 484 | 0.67 |
| SQP82655.1 | | 55 | 526 | 538 | 0.67 |
| SQP82327.1 | | 55 | 346 | 358 | 0.67 |
| MHP31245.1 | | 55 | 226 | 238 | 0.67 |
| MHP31245.1 | | 55 | 262 | 274 | 0.67 |
| MHP31245.1 | | 55 | 316 | 328 | 0.67 |
| MHP31245.1 | | 55 | 352 | 364 | 0.67 |
| MHP31245.1 | | 55 | 370 | 382 | 0.67 |
| MHP31245.1 | | 55 | 388 | 400 | 0.67 |
| MHP31245.1 | | 55 | 496 | 508 | 0.67 |
| MHP31245.1 | | 55 | 514 | 526 | 0.67 |
| MBC1114834.1 | | 55 | 238 | 250 | 0.67 |
| MBC1114834.1 | | 55 | 274 | 286 | 0.67 |
| MBC1114834.1 | | 55 | 310 | 322 | 0.67 |
| HAO2834899.1 | | 55 | 262 | 274 | 0.67 |
| HAO2834899.1 | | 55 | 280 | 292 | 0.67 |
| HAO2834899.1 | | 55 | 316 | 328 | 0.67 |
| HAO2834899.1 | | 55 | 334 | 346 | 0.67 |
| HAM0590203.1 | | 55 | 262 | 274 | 0.67 |
| HAM0068497.1 | | 55 | 160 | 172 | 0.67 |
| EGQ2049191.1 | | 55 | 184 | 196 | 0.67 |
| EGQ2049191.1 | | 55 | 418 | 430 | 0.67 |
| EGO9658688.1 | | 55 | 256 | 268 | 0.67 |
| EGM0660043.1 | | 55 | 118 | 130 | 0.67 |
| EGM0660043.1 | | 55 | 154 | 166 | 0.67 |
| EGM0660043.1 | | 55 | 208 | 220 | 0.67 |
| EGM0660043.1 | | 55 | 244 | 256 | 0.67 |
| EGM0660043.1 | | 55 | 262 | 274 | 0.67 |
| EGM0660043.1 | | 55 | 280 | 292 | 0.67 |
| EGM0660043.1 | | 55 | 388 | 400 | 0.67 |
| EGM0660043.1 | | 55 | 406 | 418 | 0.67 |
| EFO2868686.1 | | 55 | 151 | 163 | 0.67 |
| EFO2710029.1 | | 55 | 238 | 250 | 0.67 |
| EFO2710029.1 | | 55 | 328 | 340 | 0.67 |
| EFO2710029.1 | | 55 | 580 | 592 | 0.67 |
| EFO2710029.1 | | 55 | 598 | 610 | 0.67 |
| EFO2698625.1 | | 55 | 184 | 196 | 0.67 |
| EFO2507734.1 | | 55 | 43 | 55 | 0.67 |
| EFO2507734.1 | | 55 | 241 | 253 | 0.67 |
| EFO2507608.1 | | 55 | 155 | 167 | 0.67 |
| EFO2507608.1 | | 55 | 173 | 185 | 0.67 |
| EFO2507608.1 | | 55 | 227 | 239 | 0.67 |
| EFO2507608.1 | | 55 | 299 | 311 | 0.67 |
| EFO2507608.1 | | 55 | 479 | 491 | 0.67 |
| EFO2507608.1 | | 55 | 533 | 545 | 0.67 |
| EFN8303772.1 | | 55 | 100 | 112 | 0.68 |
| EFN8175469.1 | | 55 | 292 | 304 | 0.67 |
| EFN4856017.1 | | 55 | 148 | 160 | 0.67 |
| EFN4856017.1 | | 55 | 220 | 232 | 0.67 |
| EFN4856017.1 | | 55 | 274 | 286 | 0.67 |
| EFN4856017.1 | | 55 | 364 | 376 | 0.67 |
| EFN4856017.1 | | 55 | 382 | 394 | 0.67 |
| EFN4856017.1 | | 55 | 436 | 448 | 0.67 |
| EFL9638376.1 | | 55 | 75 | 87 | 0.67 |
| EFL9638376.1 | | 55 | 201 | 213 | 0.67 |
| EFH8064738.1 | | 55 | 31 | 43 | 0.67 |
| EFH8064738.1 | | 55 | 121 | 133 | 0.67 |
| EFB8895300.1 | | 55 | 129 | 141 | 0.67 |
| EFB8895300.1 | | 55 | 201 | 213 | 0.67 |
| EFB8894014.1 | | 55 | 292 | 304 | 0.67 |
| EFB8894014.1 | | 55 | 364 | 376 | 0.67 |
| EFB8894014.1 | | 55 | 400 | 412 | 0.67 |
| EFA5331929.1 | | 55 | 131 | 143 | 0.67 |
| EFA5330394.1 | | 55 | 256 | 268 | 0.67 |
| EFA5330394.1 | | 55 | 274 | 286 | 0.67 |
| EFA5330394.1 | | 55 | 328 | 340 | 0.67 |
| EFA5330394.1 | | 55 | 562 | 574 | 0.67 |
| EFA3723142.1 | | 55 | 59 | 71 | 0.67 |
| EFA3723142.1 | | 55 | 95 | 107 | 0.67 |
| EFA3723142.1 | | 55 | 113 | 125 | 0.67 |
| EFA3723142.1 | | 55 | 131 | 143 | 0.67 |
| EFA3723142.1 | | 55 | 185 | 197 | 0.67 |
| EFA3721394.1 | | 55 | 256 | 268 | 0.67 |
| EFA3721394.1 | | 55 | 274 | 286 | 0.67 |
| EEX9004507.1 | | 55 | 340 | 352 | 0.67 |
| EEU9512438.1 | | 55 | 167 | 179 | 0.68 |
| EEU9512438.1 | | 55 | 221 | 233 | 0.68 |
| EEU9512438.1 | | 55 | 239 | 251 | 0.68 |
| WP_181664680.1 | | 56 | 483 | 497 | 0.059 |
| WP_180200997.1 | | 56 | 396 | 410 | 0.059 |
| WP_175297318.1 | | 56 | 321 | 335 | 0.059 |
| WP_143362145.1 | | 56 | 381 | 395 | 0.059 |
| WP_142440302.1 | | 56 | 396 | 410 | 0.06 |
| WP_141087126.1 | | 56 | 336 | 350 | 0.059 |
| WP_140168261.1 | | 56 | 146 | 160 | 0.06 |
| WP_137441919.1 | | 56 | 336 | 350 | 0.059 |
| WP_103809109.1 | | 56 | 206 | 220 | 0.06 |
| WP_097335408.1 | | 56 | 191 | 205 | 0.06 |
| WP_096986375.1 | | 56 | 366 | 380 | 0.06 |
| WP_096986375.1 | | 56 | 206 | 219 | 0.95 |
| WP_077581230.1 | | 56 | 206 | 220 | 0.06 |
| TLD67278.1 | | 56 | 258 | 272 | 0.06 |
| TII97933.1 | | 56 | 122 | 136 | 0.06 |
| SRB30148.1 | | 56 | 336 | 350 | 0.06 |
| SPW90104.1 | | 56 | 351 | 365 | 0.059 |
| MWC58373.1 | | 56 | 321 | 335 | 0.06 |
| MHP37917.1 | | 56 | 321 | 335 | 0.06 |
| MHO94098.1 | | 56 | 366 | 380 | 0.06 |
| MBC0702481.1 | | 56 | 206 | 220 | 0.06 |
| MBB7412271.1 | | 56 | 291 | 305 | 0.06 |
| MBB0566171.1 | | 56 | 351 | 365 | 0.06 |
| HAP2426985.1 | | 56 | 366 | 380 | 0.06 |
| HAO9429127.1 | | 56 | 106 | 120 | 0.06 |
| HAO0486031.1 | | 56 | 320 | 334 | 0.06 |
| HAO0479489.1 | | 56 | 205 | 219 | 0.06 |
| HAN4783305.1 | | 56 | 257 | 271 | 0.06 |
| HAM0782593.1 | | 56 | 219 | 233 | 0.06 |
| HAM0782592.1 | | 56 | 189 | 203 | 0.06 |
| HAM0630192.1 | | 56 | 336 | 350 | 0.06 |
| HAH5465748.1 | | 56 | 291 | 305 | 0.06 |
| EGO5174632.1 | | 56 | 279 | 293 | 0.06 |
| EGO4399501.1 | | 56 | 274 | 288 | 0.06 |
| EGI2439531.1 | | 56 | 246 | 260 | 0.06 |
| EGF5301699.1 | | 56 | 336 | 350 | 0.06 |
| EFN4869179.1 | | 56 | 305 | 319 | 0.06 |
| EFM6923143.1 | | 56 | 84 | 98 | 0.06 |
| EFM0265426.1 | | 56 | 276 | 290 | 0.06 |
| EFL9446843.1 | | 56 | 206 | 220 | 0.06 |
| EFJ2531123.1 | | 56 | 219 | 233 | 0.06 |
| EFH6254796.1 | | 56 | 118 | 132 | 0.06 |
| EFH4774781.1 | | 56 | 282 | 296 | 0.06 |
| EFE7699866.1 | | 56 | 240 | 254 | 0.06 |
| EFD5400045.1 | | 56 | 366 | 380 | 0.06 |
| EFD1713145.1 | | 56 | 191 | 205 | 0.06 |
| EFB9623657.1 | | 56 | 366 | 380 | 0.06 |
| EEW1561384.1 | | 56 | 411 | 425 | 0.059 |
| WP_172480638.1 | | 57 | 163 | 177 | 0.042 |
| WP_172480638.1 | | 58 | 169 | 182 | 0.011 |
| WP_158700857.1 | | 111 | 30 | 42 | 0.004 |
| WP_158700857.1 | | 117 | 18 | 32 | 4.95e-04 |
| WP_158700857.1 | | 148 | 62 | 75 | 0.006 |
| WP_152961091.1 | | 153 | 198 | 216 | 1.08e-10 |
| WP_152931027.1 | | 153 | 43 | 61 | 1.09e-10 |
| WP_152921976.1 | | 153 | 161 | 179 | 1.08e-10 |
| WP_176232723.1 | | 156 | 116 | 135 | 2.52e-05 |
| WP_152961079.1 | | 156 | 113 | 132 | 3.68e-08 |
| WP_152922000.1 | | 156 | 48 | 67 | 3.74e-08 |
| WP_126755780.1 | | 156 | 433 | 452 | 9.97e-05 |
| WP_126755780.1 | | 156 | 433 | 452 | 9.97e-05 |
| WP_152961091.1 | | 157 | 183 | 202 | 1.85e-08 |
| WP_152931027.1 | | 157 | 28 | 47 | 1.87e-08 |
| WP_152921976.1 | | 157 | 146 | 165 | 1.86e-08 |
| WP_176232855.1 | | 159 | 30 | 48 | 4.79e-09 |
| WP_176232680.1 | | 159 | 30 | 48 | 7.27e-08 |
| WP_152961091.1 | | 159 | 3 | 20 | 1.68e-09 |
| WP_152952033.1 | | 159 | 30 | 48 | 4.73e-09 |
| WP_152928580.1 | | 159 | 30 | 48 | 4.74e-09 |
| WP_152921974.1 | | 159 | 56 | 73 | 1.68e-09 |
| WP_126755780.1 | | 159 | 26 | 44 | 4.65e-09 |
| WP_152953065.1 | | 160 | 7 | 26 | 1.80e-05 |
| WP_152952148.1 | | 160 | 32 | 51 | 1.81e-05 |
| WP_152932209.1 | | 160 | 21 | 40 | 1.80e-05 |
| MQL41398.1 | | 160 | 21 | 40 | 1.80e-05 |
| WP_152961091.1 | | 161 | 153 | 172 | 1.04e-07 |
| WP_152921974.1 | | 161 | 206 | 222 | 2.54e-05 |
| WP_152921531.1 | | 161 | 24 | 43 | 1.04e-07 |
| WP_152961091.1 | | 163 | 243 | 262 | 5.77e-07 |
| WP_152931027.1 | | 163 | 88 | 107 | 5.80e-07 |
| WP_152961079.1 | | 164 | 158 | 177 | 1.68e-09 |
| WP_152931025.1 | | 164 | 32 | 51 | 1.69e-09 |
| WP_176232689.1 | | 165 | 64 | 83 | 6.59e-09 |
| WP_176232680.1 | | 165 | 197 | 216 | 6.58e-09 |
| WP_152961125.1 | | 165 | 19 | 38 | 6.79e-09 |
| WP_152961091.1 | | 165 | 168 | 187 | 1.32e-08 |
| WP_152947140.1 | | 165 | 3 | 22 | 6.81e-09 |
| WP_152931027.1 | | 165 | 13 | 32 | 1.33e-08 |
| WP_152921531.1 | | 165 | 39 | 58 | 2.65e-08 |
| WP_152961079.1 | | 167 | 38 | 57 | 2.14e-10 |
| WP_152932471.1 | | 167 | 48 | 67 | 2.10e-07 |
| WP_152931027.1 | | 167 | 178 | 197 | 2.16e-10 |
| WP_152930401.1 | | 167 | 36 | 55 | 2.20e-10 |
| WP_126755780.1 | | 167 | 356 | 375 | 7.09e-05 |
| WP_176232855.1 | | 169 | 15 | 34 | 2.55e-05 |
| WP_176232680.1 | | 169 | 15 | 34 | 2.53e-05 |
| WP_152952033.1 | | 169 | 15 | 34 | 2.54e-05 |
| WP_152928580.1 | | 169 | 15 | 34 | 2.54e-05 |
| WP_152921974.1 | | 169 | 41 | 60 | 3.04e-10 |
| WP_126755780.1 | | 169 | 11 | 30 | 1.45e-07 |
| WP_152961119.1 | | 170 | 21 | 40 | 1.56e-10 |
| WP_152961079.1 | | 170 | 83 | 102 | 1.86e-08 |
| WP_152932471.1 | | 170 | 95 | 111 | 5.32e-08 |
| WP_152930401.1 | | 170 | 81 | 100 | 1.90e-08 |
| WP_152922000.1 | | 170 | 18 | 37 | 1.89e-08 |
| WP_152961091.1 | | 172 | 228 | 247 | 1.86e-08 |
| WP_152931027.1 | | 172 | 73 | 92 | 1.87e-08 |
| WP_152921976.1 | | 172 | 191 | 210 | 1.87e-08 |
| WP_152961079.1 | | 175 | 173 | 192 | 4.25e-10 |
| WP_152931025.1 | | 175 | 47 | 66 | 4.30e-10 |
| WP_176232855.1 | | 176 | 59 | 78 | 7.14e-05 |
| WP_176232680.1 | | 176 | 59 | 78 | 5.03e-05 |
| WP_152961091.1 | | 176 | 34 | 53 | 1.86e-08 |
| WP_152952033.1 | | 176 | 59 | 78 | 5.05e-05 |
| WP_152932649.1 | | 176 | 5 | 24 | 7.16e-05 |
| WP_152928580.1 | | 176 | 59 | 78 | 5.05e-05 |
| WP_152921974.1 | | 176 | 87 | 106 | 1.87e-08 |
| WP_126755780.1 | | 176 | 55 | 74 | 5.03e-05 |
| WP_152961079.1 | | 179 | 188 | 207 | 1.19e-09 |
| WP_152931025.1 | | 179 | 77 | 96 | 1.20e-09 |
| WP_152961079.1 | | 180 | 128 | 147 | 1.27e-05 |
| WP_152931025.1 | | 180 | 2 | 21 | 1.28e-05 |
| WP_152922000.1 | | 180 | 63 | 82 | 1.28e-05 |
| WP_152931027.1 | | 183 | 133 | 152 | 2.92e-07 |
| WP_152961091.1 | | 184 | 213 | 232 | 5.21e-08 |
| WP_152931027.1 | | 184 | 58 | 77 | 5.24e-08 |
| WP_152921976.1 | | 184 | 176 | 195 | 5.24e-08 |
| WP_176232689.1 | | 187 | 169 | 184 | 1.41e-04 |
| WP_176232680.1 | | 187 | 302 | 317 | 1.79e-05 |
| WP_152961091.1 | | 187 | 273 | 292 | 6.00e-10 |
| WP_152952160.1 | | 187 | 5 | 20 | 1.81e-05 |
| WP_152931064.1 | | 187 | 92 | 107 | 1.81e-05 |
| WP_152931027.1 | | 187 | 118 | 137 | 6.05e-10 |
| WP_152930481.1 | | 187 | 13 | 28 | 1.82e-05 |
| WP_152921531.1 | | 187 | 146 | 162 | 0.006 |
| WP_152961079.1 | | 188 | 23 | 42 | 4.25e-10 |
| WP_152931027.1 | | 188 | 163 | 182 | 4.29e-10 |
| WP_152930401.1 | | 188 | 21 | 40 | 4.37e-10 |
| WP_152961079.1 | | 189 | 219 | 237 | 3.69e-08 |
| WP_152931025.1 | | 189 | 108 | 126 | 3.72e-08 |
| WP_152961079.1 | | 191 | 98 | 117 | 3.69e-08 |
| WP_152930401.1 | | 191 | 96 | 115 | 3.77e-08 |
| WP_152922000.1 | | 191 | 33 | 52 | 3.75e-08 |
| *Escherichia coli O19* | | | | | |
| EFA4307993.1 | | 55 | 17 | 29 | 0.68 |
| EFA4307993.1 | | 55 | 71 | 83 | 0.68 |
| EFA4307993.1 | | 55 | 107 | 119 | 0.68 |
| EFA4307993.1 | | 55 | 125 | 137 | 0.68 |
| EFA4307993.1 | | 55 | 161 | 173 | 0.68 |
| *Escherichia coli O43:H14* | | | | | |
| EFA4176382.1 | | 55 | 184 | 196 | 0.67 |
| EFA4176382.1 | | 55 | 256 | 268 | 0.67 |
| EFA4176382.1 | | 55 | 292 | 304 | 0.67 |
| *Escherichia coli O78:H42* | | | | | |
| EFA4141733.1 | | 55 | 221 | 233 | 0.67 |
| *Escherichia coli O17* | | | | | |
| EEZ8624652.1 | | 56 | 279 | 293 | 0.06 |
| *Klebsiella pneumoniae* | | | | | |
| WP_159188171.1 | | 48 | 65 | 78 | 0.004 |
| WP_159316061.1 | | 48 | 35 | 49 | 2.42e-04 |
| WP_139109296.1 | | 49 | 41 | 56 | 0.17 |
| WP_172488678.1 | | 49 | 97 | 112 | 0.002 |
| WP_163540424.1 | | 55 | 44 | 58 | 0.49 |
| OCR48604.1 | | 76 | 25 | 43 | 0.005 |
| WP_172718143.1 | | 104 | 151 | 163 | 0.003 |
| WP_172718143.1 | | 105 | 136 | 150 | 3.02e-05 |
| WP_172718143.1 | | 112 | 149 | 163 | 9.64e-07 |
| WP_172718143.1 | | 145 | 181 | 193 | 1.52e-05 |
| WP_172718143.1 | | 146 | 149 | 163 | 9.64e-07 |
| KAB1671898.1 | | 152 | 115 | 133 | 5.16e-08 |
| KAB1671898.1 | | 159 | 26 | 44 | 5.72e-07 |
| KAB1705422.1 | | 159 | 30 | 48 | 7.41e-08 |
| WP_159122356.1 | | 159 | 26 | 44 | 4.76e-09 |
| KAB1705560.1 | | 160 | 18 | 37 | 9.07e-06 |
| KAB1671898.1 | | 161 | 174 | 194 | 3.20e-06 |
| KAB1671898.1 | | 165 | 190 | 209 | 7.30e-08 |
| KAB1705731.1 | | 165 | 39 | 58 | 6.67e-09 |
| WP_159306668.1 | | 165 | 37 | 56 | 6.74e-09 |
| KAB1671898.1 | | 167 | 355 | 374 | 2.60e-08 |
| WP_151465977.1 | | 167 | 102 | 121 | 2.62e-08 |
| KAB1705422.1 | | 169 | 15 | 34 | 2.54e-05 |
| WP_159122356.1 | | 169 | 11 | 30 | 2.08e-07 |
| KAB1671898.1 | | 170 | 115 | 133 | 5.16e-08 |
| WP_151465977.1 | | 170 | 150 | 169 | 2.90e-07 |
| WP_175282747.1 | | 175 | 1 | 15 | 0.54 |
| KAB1705422.1 | | 176 | 59 | 78 | 5.06e-05 |
| WP_159122356.1 | | 176 | 55 | 74 | 7.13e-05 |
| KAB1671898.1 | | 180 | 450 | 467 | 3.56e-05 |
| WP_151465977.1 | | 180 | 197 | 214 | 3.57e-05 |
| KAB1705731.1 | | 187 | 144 | 159 | 1.80e-05 |
| WP_159306671.1 | | 187 | 1 | 12 | 0.025 |
| WP_159314147.1 | | 193 | 79 | 90 | 0.77 |
| OCR48604.1 | | 205 | 29 | 41 | 0.002 |
| KAA5754462.1 | | 209 | 46 | 60 | 0.011 |
| KAB1717638.1 | | 211 | 107 | 121 | 3.03e-05 |
| KAB1717638.1 | | 217 | 37 | 51 | 0.011 |
| *Mycobacterium tuberculosis* | | | | | |
| WP_105815007.1 | | 99 | 54 | 68 | 5.29e-06 |
| WP_105815007.1 | | 102 | 206 | 220 | 7.47e-06 |
| WP_105815007.1 | | 103 | 120 | 134 | 1.88e-06 |
| WP_105815007.1 | | 104 | 439 | 453 | 5.99e-08 |
| WP_105815007.1 | | 105 | 424 | 438 | 5.99e-08 |
| WP_105815007.1 | | 106 | 214 | 228 | 4.74e-07 |
| WP_105815007.1 | | 108 | 64 | 78 | 7.47e-06 |
| WP_105815007.1 | | 111 | 285 | 299 | 3.75e-06 |
| WP_105815007.1 | | 112 | 437 | 451 | 1.51e-08 |
| WP_105815007.1 | | 113 | 478 | 492 | 3.81e-09 |
| WP_105815007.1 | | 114 | 72 | 86 | 5.92e-05 |
| WP_105815007.1 | | 117 | 274 | 288 | 8.46e-08 |
| WP_105815007.1 | | 118 | 219 | 233 | 1.68e-07 |
| WP_105815007.1 | | 124 | 200 | 213 | 8.36e-05 |
| WP_105815007.1 | | 125 | 421 | 434 | 8.36e-05 |
| WP_105815007.1 | | 128 | 394 | 408 | 5.99e-08 |
| WP_105815007.1 | | 129 | 192 | 206 | 5.29e-06 |
| WP_105815007.1 | | 133 | 223 | 237 | 3.01e-08 |
| WP_105815007.1 | | 139 | 229 | 243 | 3.75e-06 |
| WP_105815007.1 | | 141 | 364 | 378 | 2.65e-06 |
| WP_105815007.1 | | 145 | 469 | 483 | 2.70e-09 |
| WP_105815007.1 | | 148 | 319 | 333 | 1.19e-07 |
| WP_105815007.1 | | 200 | 74 | 85 | 0.034 |
| *Nocardia farcinica* | | | | | |
| WP_195025737.1 | | 165 | 62 | 81 | 6.80e-09 |
| *Prevotella copri* | | | | | |
| WP_006848473.1 | | 186 | 382 | 400 | 1.41e-04 |
| *Prevotella sp. CAG:255* | | | | | |
| CCX68624.1 | | 186 | 382 | 400 | 1.41e-04 |
| *Prevotella sp. KH2C16* | | | | | |
| WP_092114275.1 | | 186 | 381 | 399 | 1.41e-04 |
| *Prevotella sp. oral taxon 820* | | | | | |
| WP_107581795.1 | | 186 | 382 | 400 | 1.41e-04 |
| *Proteus mirabilis* | | | | | |
| WP_171948676.1 | | 48 | 114 | 127 | 0.34 |
| *Ruthenibacterium lactatiformans* | | | | | |
| WP_172726351.1 | | 191 | 387 | 405 | 2.89e-07 |
| WP_172726658.1 | | 191 | 387 | 405 | 2.89e-07 |
| WP_172729695.1 | | 191 | 387 | 405 | 2.89e-07 |
| MBD9256670.1 | | 191 | 387 | 405 | 2.89e-07 |
| WP_172734110.1 | | 191 | 387 | 405 | 2.89e-07 |
| *Staphylococcus haemolyticus* | | | | | |
| WP_180553187.1 | | 6 | 42 | 56 | 0.062 |
| WP_180553187.1 | | 91 | 36 | 44 | 0.46 |
| *Streptococcus pyogenes* | | | | | |
| PZO93090.1 | | 56 | 153 | 167 | 0.021 |
| PZO93090.1 | | 56 | 81 | 95 | 0.67 |
| PZO95822.1 | | 56 | 110 | 124 | 0.011 |
| QCK38000.1 | | 56 | 130 | 144 | 6.64e-04 |
| QCK38000.1 | | 56 | 178 | 192 | 6.64e-04 |
| VGU78002.1 | | 56 | 209 | 223 | 0.021 |
| VHB81408.1 | | 56 | 88 | 102 | 0.021 |
| VHE43714.1 | | 56 | 87 | 101 | 0.021 |
| VHE43714.1 | | 56 | 135 | 149 | 0.021 |
| VHF44597.1 | | 56 | 87 | 101 | 0.021 |
| VHF44597.1 | | 56 | 135 | 149 | 0.021 |
| VHF49783.1 | | 56 | 88 | 102 | 0.021 |
| VHG74531.1 | | 56 | 87 | 101 | 0.021 |
| VHG74531.1 | | 56 | 135 | 149 | 0.021 |
| VHM87233.1 | | 56 | 87 | 101 | 0.021 |
| WP_093974700.1 | | 56 | 128 | 142 | 0.021 |
| WP_093974700.1 | | 56 | 176 | 190 | 0.021 |
| WP_136132813.1 | | 56 | 88 | 102 | 0.021 |
| WP_136286420.1 | | 56 | 6 | 20 | 0.021 |
| WP_136291992.1 | | 56 | 173 | 187 | 0.021 |
| WP_136297990.1 | | 56 | 6 | 20 | 0.021 |
| WP_136297995.1 | | 56 | 113 | 127 | 0.021 |
| WP_136302243.1 | | 56 | 234 | 248 | 0.021 |
| WP_136304062.1 | | 56 | 6 | 20 | 0.021 |
| WP_168642164.1 | | 56 | 70 | 84 | 0.021 |
| WP_168642164.1 | | 56 | 118 | 132 | 0.021 |
| WP_168644371.1 | | 56 | 70 | 84 | 0.021 |
| WP_168671222.1 | | 56 | 101 | 115 | 0.021 |
| WP_168671222.1 | | 56 | 149 | 163 | 0.021 |
| WP_168693887.1 | | 56 | 70 | 84 | 0.021 |
| WP_168693887.1 | | 56 | 118 | 132 | 0.021 |
| WP_186799892.1 | | 56 | 169 | 183 | 6.64e-04 |
| WP_186799892.1 | | 56 | 217 | 231 | 6.64e-04 |
| WP_194073975.1 | | 56 | 85 | 99 | 6.67e-04 |
| WP_194091732.1 | | 56 | 208 | 222 | 0.021 |
| WP_194104795.1 | | 56 | 70 | 84 | 0.021 |
