# Supplementary material for: Mining the capacity of human-associated microorganisms to trigger rheumatoid arthritis—A systematic immunoinformatics analysis of T cell epitopes
Source: PLoS One. 2021 Jun 29;16(6):e0253918. doi: 10.1371/journal.pone.0253918 (PMC8241107; doi:10.1371/journal.pone.0253918)
Supplement: S6 Table — (DOCX) [file pone.0253918.s006.docx]

Mining the capacity of human-associated microorganisms to trigger rheumatoid arthritis – a systematic immunoinformatics analysis of T cell epitopes

Jelena Repac^1^, Marija Mandić^1^, Tanja Lunić^1^, Bojan Božić^1*¶^, Biljana Božić Nedeljković^1*¶^

^1^ Institute of Physiology and Biochemistry “Ivan Djaja”, Faculty of Biology, University of Belgrade, Belgrade, Serbia

# **S6 Table**. **The distribution of BLASTp hits across fungi human pathogen/commensals where the relation between rheumatoid arthritis and the corresponding species has not been previously established in literature (PubMed).**

| Fungi | | | | | |
| --- | --- | --- | --- | --- | --- |
| Accession Number | **Epitope Number** | | **Start** | **Stop** | **e value** |
| *Absidia glauca* | | | | | |
| SAM08991.1 | | 159 | 135 | 153 | 2.03e-10 |
| SAM01991.1 | | 169 | 41 | 60 | 4.48e-09 |
| SAM08991.1 | | 169 | 120 | 139 | 4.48e-09 |
| SAM08991.1 | | 170 | 509 | 528 | 4.04e-10 |
| SAM08991.1 | | 190 | 253 | 272 | 3.29e-12 |
| SAM01991.1 | | 191 | 445 | 464 | 4.04e-10 |
| SAM08991.1 | | 191 | 524 | 543 | 4.04e-10 |
| *Absidia repens* | | | | | |
| ORZ23613.1 | | 169 | 41 | 60 | 4.48e-09 |
| ORZ25547.1 | | 169 | 41 | 60 | 4.48e-09 |
| ORZ23613.1 | | 191 | 445 | 464 | 4.04e-10 |
| ORZ25547.1 | | 191 | 445 | 464 | 4.04e-10 |
| *Apiotrichum porosum* | | | | | |
| XP_028480718.1 | | 158 | 49 | 68 | 1.73e-05 |
| XP_028477643.1 | | 161 | 173 | 193 | 2.50e-08 |
| XP_028480718.1 | | 167 | 408 | 427 | 2.87e-10 |
| XP_028480718.1 | | 188 | 393 | 412 | 1.13e-09 |
| *Apophysomyces ossiformis* | | | | | |
| KAF7722541.1 | | 169 | 44 | 63 | 4.48e-09 |
| KAF7722541.1 | | 191 | 447 | 466 | 4.04e-10 |
| KAF7724475.1 | | 210 | 1 | 15 | 8.14e-05 |
| KAF7721657.1 | | 222 | 26 | 40 | 3.24e-04 |
| *Aureobasidium melanogenum CBS 110374* | | | | | |
| KEQ60096.1 | | 153 | 262 | 280 | 4.48e-09 |
| KEQ60096.1 | | 178 | 367 | 386 | 1.02e-10 |
| *Aureobasidium namibiae CBS 147.97* | | | | | |
| XP_013429338.1 | | 34 | 186 | 196 | 0.035 |
| *Aureobasidium pullulans* | | | | | |
| OBW67097.1 | | 5 | 195 | 204 | 0.25 |
| THV73698.1 | | 5 | 195 | 204 | 0.25 |
| THV94641.1 | | 5 | 195 | 204 | 0.25 |
| THW23905.1 | | 5 | 195 | 204 | 0.25 |
| THW56630.1 | | 5 | 195 | 204 | 0.25 |
| THW57648.1 | | 5 | 195 | 204 | 0.25 |
| THW85218.1 | | 5 | 195 | 204 | 0.25 |
| THW99507.1 | | 5 | 195 | 204 | 0.25 |
| THX23438.1 | | 5 | 195 | 204 | 0.25 |
| THX36668.1 | | 5 | 195 | 204 | 0.25 |
| THY23044.1 | | 5 | 195 | 204 | 0.25 |
| THY56977.1 | | 5 | 195 | 204 | 0.25 |
| THY58872.1 | | 5 | 195 | 204 | 0.25 |
| THY72014.1 | | 5 | 195 | 204 | 0.25 |
| THZ00712.1 | | 5 | 195 | 204 | 0.25 |
| THZ02597.1 | | 5 | 195 | 204 | 0.25 |
| THZ26911.1 | | 5 | 195 | 204 | 0.25 |
| THZ78030.1 | | 5 | 195 | 204 | 0.25 |
| THZ81064.1 | | 5 | 195 | 204 | 0.25 |
| TIA12528.1 | | 5 | 195 | 204 | 0.25 |
| TIA21683.1 | | 5 | 195 | 204 | 0.25 |
| TIA31249.1 | | 5 | 195 | 204 | 0.25 |
| THV74046.1 | | 153 | 282 | 300 | 4.48e-09 |
| THV83749.1 | | 153 | 261 | 279 | 4.48e-09 |
| THW30161.1 | | 153 | 281 | 299 | 4.48e-09 |
| THX18765.1 | | 153 | 261 | 279 | 4.48e-09 |
| THX30611.1 | | 153 | 261 | 279 | 4.48e-09 |
| THX81467.1 | | 153 | 261 | 279 | 4.48e-09 |
| THX89591.1 | | 153 | 282 | 300 | 4.48e-09 |
| THY36675.1 | | 153 | 312 | 330 | 4.48e-09 |
| THZ04339.1 | | 153 | 261 | 279 | 4.48e-09 |
| THZ78977.1 | | 153 | 261 | 279 | 4.48e-09 |
| TIA14862.1 | | 153 | 282 | 300 | 4.48e-09 |
| TIA74585.1 | | 153 | 282 | 300 | 4.48e-09 |
| THV74046.1 | | 178 | 387 | 406 | 1.02e-10 |
| THV83749.1 | | 178 | 366 | 385 | 1.02e-10 |
| THW30161.1 | | 178 | 386 | 405 | 1.02e-10 |
| THX30611.1 | | 178 | 366 | 385 | 1.02e-10 |
| THX81467.1 | | 178 | 366 | 385 | 1.02e-10 |
| THX89591.1 | | 178 | 387 | 406 | 1.02e-10 |
| THY36675.1 | | 178 | 417 | 436 | 1.02e-10 |
| THZ04339.1 | | 178 | 366 | 385 | 1.02e-10 |
| THZ78977.1 | | 178 | 366 | 385 | 1.02e-10 |
| TIA14862.1 | | 178 | 387 | 406 | 1.02e-10 |
| TIA74585.1 | | 178 | 387 | 406 | 1.02e-10 |
| *Aureobasidium pullulans eXF-150* | | | | | |
| XP_029762874.1 | | 5 | 195 | 204 | 0.25 |
| XP_029763370.1 | | 153 | 261 | 279 | 4.48e-09 |
| XP_029763370.1 | | 178 | 366 | 385 | 1.02e-10 |
| *Blastomyces gilchristii SLH14081* | | | | | |
| XP_002627459.1 | | 158 | 46 | 62 | 1.73e-05 |
| XP_002627459.1 | | 180 | 491 | 510 | 1.40e-07 |
| *Blastomyces parvus* | | | | | |
| PGH09182.1 | | 158 | 46 | 62 | 1.73e-05 |
| PGH09182.1 | | 180 | 491 | 510 | 1.40e-07 |
| *Blastomyces percursus* | | | | | |
| OJD22326.1 | | 158 | 46 | 62 | 1.73e-05 |
| OJD22326.1 | | 180 | 491 | 510 | 1.40e-07 |
| *Byssochlamys spectabilis* | | | | | |
| XP_028489444.1 | | 153 | 262 | 281 | 2.25e-09 |
| *Byssochlamys spectabilis No. 5* | | | | | |
| GAD92595.1 | | 34 | 208 | 219 | 0.012 |
| GAD96110.1 | | 153 | 262 | 281 | 2.25e-09 |
| *Cladophialophora bantiana CBS 173.52* | | | | | |
| XP_016620036.1 | | 159 | 79 | 97 | 2.03e-10 |
| XP_016620036.1 | | 185 | 183 | 201 | 1.02e-10 |
| XP_016620036.1 | | 190 | 197 | 216 | 3.30e-12 |
| *Cladophialophora carrionii CBS 160.54* | | | | | |
| XP_008731153.1 | | 159 | 79 | 97 | 2.03e-10 |
| XP_008726381.1 | | 176 | 54 | 73 | 5.53e-07 |
| XP_008731153.1 | | 185 | 183 | 201 | 1.02e-10 |
| XP_008731153.1 | | 190 | 197 | 216 | 3.30e-12 |
| *Cladophialophora immunda* | | | | | |
| OQV09414.1 | | 159 | 79 | 97 | 2.03e-10 |
| XP_016242368.1 | | 159 | 79 | 97 | 2.03e-10 |
| OQV09414.1 | | 185 | 183 | 201 | 1.02e-10 |
| XP_016242368.1 | | 185 | 183 | 201 | 1.02e-10 |
| OQV09414.1 | | 190 | 197 | 216 | 3.30e-12 |
| XP_016242368.1 | | 190 | 197 | 216 | 3.30e-12 |
| *Cladosporium cladosporioides* | | | | | |
| AET14829.1 | | 165 | 189 | 208 | 5.70e-10 |
| *Claviceps purpurea 20.1* | | | | | |
| CCE31680.1 | | 5 | 619 | 628 | 0.25 |
| CCE27207.1 | | 182 | 1149 | 1168 | 2.25e-09 |
| CCE28306.1 | | 220 | 59 | 70 | 0.029 |
| *Colletotrichum tofieldiae* | | | | | |
| KZL76229.1 | | 180 | 568 | 586 | 2.78e-07 |
| *Conidiobolus coronatus NRRL 28638* | | | | | |
| KXN69138.1 | | 182 | 306 | 325 | 8.04e-10 |
| KXN69138.1 | | 185 | 156 | 175 | 9.24e-12 |
| *Cyberlindnera fabianii* | | | | | |
| ONH66793.1 | | 165 | 494 | 513 | 5.69e-10 |
| CDR39394.1 | | 186 | 423 | 438 | 1.10e-06 |
| ONH65602.1 | | 186 | 203 | 218 | 1.10e-06 |
| *Cyphellophora europaea CBS 101466* | | | | | |
| XP_008718524.1 | | 158 | 44 | 59 | 1.55e-06 |
| XP_008718524.1 | | 159 | 70 | 88 | 2.03e-10 |
| XP_008718524.1 | | 164 | 518 | 536 | 2.19e-06 |
| XP_008718524.1 | | 170 | 443 | 462 | 7.25e-11 |
| XP_008718524.1 | | 179 | 549 | 567 | 3.92e-07 |
| *Emergomyces pasteurianus ep9510* | | | | | |
| OJD10112.1 | | 158 | 46 | 62 | 1.73e-05 |
| OJD10112.1 | | 180 | 491 | 510 | 1.40e-07 |
| *Emmonsia crescens* | | | | | |
| PGH29521.1 | | 103 | 611 | 619 | 0.47 |
| PGH33907.1 | | 180 | 491 | 510 | 1.40e-07 |
| *Emmonsia crescens UAMH 3008* | | | | | |
| KKZ68235.1 | | 180 | 525 | 544 | 1.40e-07 |
| *Emmonsia sp. CAC-2015a* | | | | | |
| OAX77767.1 | | 180 | 425 | 444 | 1.40e-07 |
| *Galerina marginata CBS 339.88* | | | | | |
| KDR74124.1 | | 167 | 398 | 417 | 2.87e-10 |
| KDR74124.1 | | 186 | 428 | 447 | 2.19e-06 |
| KDR74124.1 | | 187 | 338 | 357 | 6.55e-12 |
| KDR85514.1 | | 201 | 11 | 25 | 1.82e-06 |
| KDR85514.1 | | 210 | 1 | 15 | 8.14e-05 |
| *Hortaea werneckii* | | | | | |
| RMY91174.1 | | 178 | 381 | 400 | 8.04e-10 |
| RMY85872.1 | | 178 | 381 | 400 | 8.04e-10 |
| *Kwoniella pini CBS 10737* | | | | | |
| XP_019012960.1 | | 152 | 165 | 183 | 6.55e-12 |
| XP_019012960.1 | | 158 | 46 | 65 | 2.78e-07 |
| XP_019012960.1 | | 167 | 405 | 424 | 2.87e-10 |
| XP_019012960.1 | | 176 | 105 | 124 | 2.50e-08 |
| XP_019012960.1 | | 187 | 345 | 364 | 6.55e-12 |
| XP_019012960.1 | | 188 | 390 | 409 | 1.60e-09 |
| *Lasiodiplodia theobromae* | | | | | |
| XP_035371888.1 | | 178 | 368 | 387 | 1.02e-10 |
| *Lichtheimia corymbifera JMRC:FSU:9682* | | | | | |
| CDH60531.1 | | 169 | 45 | 64 | 4.48e-09 |
| CDH60531.1 | | 191 | 448 | 467 | 4.04e-10 |
| CDH55278.1 | | 222 | 27 | 41 | 0.003 |
| *Lichtheimia ramosa* | | | | | |
| CDS07402.1 | | 169 | 45 | 64 | 4.48e-09 |
| CDS07402.1 | | 191 | 448 | 467 | 4.04e-10 |
| *Lodderomyces elongisporus NRRL YB-4239* | | | | | |
| XP_001528644.1 | | 190 | 190 | 209 | 3.30e-12 |
| *Macrophomina phaseolina MS6* | | | | | |
| EKG12768.1 | | 178 | 368 | 387 | 1.02e-10 |
| *Metarhizium anisopliae BRIP 53293* | | | | | |
| KJK74257.1 | | 176 | 57 | 76 | 2.25e-09 |
| *Metarhizium brunneum* | | | | | |
| QLI74174.1 | | 220 | 78 | 89 | 0.66 |
| *Metarhizium brunneum ARSeF 3297* | | | | | |
| XP_014545163.1 | | 176 | 57 | 76 | 2.25e-09 |
| XP_014543878.1 | | 220 | 59 | 70 | 0.66 |
| *Metarhizium guizhouense ARSeF 977* | | | | | |
| KID91460.1 | | 176 | 57 | 76 | 2.25e-09 |
| *Metarhizium robertsii* | | | | | |
| EXV01178.1 | | 220 | 59 | 70 | 0.66 |
| *Metarhizium robertsii ARSeF 23* | | | | | |
| XP_007823896.1 | | 176 | 57 | 76 | 2.25e-09 |
| XP_007818331.1 | | 180 | 480 | 497 | 2.77e-07 |
| XP_007818331.1 | | 190 | 180 | 199 | 3.28e-12 |
| XP_007816419.1 | | 220 | 59 | 70 | 0.66 |
| *Microsporum canis CBS 113480* | | | | | |
| XP_002847153.1 | | 170 | 445 | 464 | 4.04e-10 |
| *Millerozyma farinosa CBS 7064* | | | | | |
| CCE88401.1 | | 159 | 69 | 88 | 1.84e-11 |
| CCE82193.1 | | 159 | 69 | 88 | 1.84e-11 |
| *Moesziomyces antarcticus* | | | | | |
| SPO48635.1 | | 5 | 112 | 121 | 0.25 |
| SPO48635.1 | | 5 | 116 | 125 | 0.25 |
| SPO48635.1 | | 5 | 120 | 129 | 0.25 |
| SPO48635.1 | | 5 | 124 | 133 | 0.25 |
| XP_014654326.1 | | 5 | 112 | 121 | 0.25 |
| XP_014654326.1 | | 5 | 116 | 125 | 0.25 |
| XP_014654326.1 | | 5 | 120 | 129 | 0.25 |
| XP_014654326.1 | | 5 | 124 | 133 | 0.25 |
| SPO43661.1 | | 159 | 89 | 108 | 9.24e-12 |
| XP_014658722.1 | | 159 | 484 | 503 | 9.20e-12 |
| SPO43661.1 | | 170 | 460 | 479 | 4.04e-10 |
| XP_014658722.1 | | 170 | 855 | 874 | 4.03e-10 |
| SPO43661.1 | | 187 | 356 | 375 | 6.55e-12 |
| XP_014658722.1 | | 187 | 751 | 770 | 6.53e-12 |
| SPO43661.1 | | 190 | 206 | 225 | 3.30e-12 |
| XP_014658722.1 | | 190 | 601 | 620 | 3.28e-12 |
| XP_014656973.1 | | 222 | 26 | 40 | 4.58e-04 |
| *Moesziomyces antarcticus T-34* | | | | | |
| GAC72805.1 | | 159 | 534 | 553 | 9.20e-12 |
| GAC74165.1 | | 165 | 271 | 290 | 5.70e-10 |
| GAC72805.1 | | 170 | 905 | 924 | 4.03e-10 |
| GAC72805.1 | | 187 | 801 | 820 | 6.53e-12 |
| GAC72805.1 | | 190 | 651 | 670 | 3.28e-12 |
| *Moesziomyces aphidis DSM 70725* | | | | | |
| ETS61195.1 | | 83 | 124 | 134 | 0.79 |
| ETS61164.1 | | 159 | 460 | 479 | 9.20e-12 |
| ETS61164.1 | | 170 | 841 | 860 | 4.03e-10 |
| ETS61164.1 | | 187 | 737 | 756 | 6.53e-12 |
| ETS61164.1 | | 190 | 577 | 596 | 3.28e-12 |
| ETS59920.1 | | 222 | 26 | 40 | 4.58e-04 |
| *Nannizzia gypsea CBS 118893* | | | | | |
| XP_003169808.1 | | 170 | 445 | 464 | 4.04e-10 |
| *Ophiostoma piceae UAMH 11346* | | | | | |
| EPE03626.1 | | 58 | 460 | 470 | 0.47 |
| *Paxillus involutus ATCC 200175* | | | | | |
| KIJ09930.1 | | 201 | 11 | 25 | 1.82e-06 |
| KIJ09930.1 | | 222 | 26 | 40 | 1.15e-04 |
| *Phialemoniopsis curvata* | | | | | |
| XP_030991972.1 | | 182 | 316 | 335 | 2.25e-09 |
| *Piedraia hortae CBS 480.64* | | | | | |
| KAF2860610.1 | | 154 | 191 | 210 | 3.65e-11 |
| KAF2860610.1 | | 178 | 356 | 375 | 1.02e-10 |
| *Purpureocillium lilacinum* | | | | | |
| PWI75594.1 | | 157 | 241 | 260 | 1.39e-07 |
| PWI75594.1 | | 180 | 482 | 500 | 2.77e-07 |
| *Rhinocladiella mackenziei CBS 650.93* | | | | | |
| XP_013271093.1 | | 4 | 1090 | 1099 | 1 |
| XP_013271086.1 | | 153 | 266 | 285 | 1.26e-08 |
| XP_013271086.1 | | 170 | 445 | 464 | 7.25e-11 |
| XP_013276077.1 | | 176 | 73 | 92 | 5.53e-07 |
| XP_013271086.1 | | 180 | 490 | 509 | 1.77e-08 |
| *Rhizoctonia solani* | | | | | |
| CUA67663.1 | | 152 | 238 | 256 | 6.55e-12 |
| CUA70581.1 | | 152 | 151 | 169 | 6.54e-12 |
| KAF8679523.1 | | 152 | 151 | 169 | 6.55e-12 |
| KAF8707467.1 | | 152 | 151 | 169 | 6.55e-12 |
| KAF8761241.1 | | 152 | 151 | 169 | 6.56e-12 |
| CUA67663.1 | | 185 | 252 | 271 | 9.24e-12 |
| CUA70581.1 | | 185 | 165 | 184 | 9.22e-12 |
| KAF8679523.1 | | 185 | 165 | 184 | 9.24e-12 |
| KAF8707467.1 | | 185 | 165 | 184 | 9.24e-12 |
| KAF8761241.1 | | 185 | 165 | 184 | 9.24e-12 |
| CUA67663.1 | | 190 | 267 | 286 | 3.30e-12 |
| CUA70581.1 | | 190 | 180 | 199 | 3.29e-12 |
| CUA75463.1 | | 190 | 400 | 419 | 3.29e-12 |
| CUA70581.1 | | 191 | 657 | 676 | 7.24e-11 |
| KAF8679523.1 | | 191 | 449 | 468 | 7.26e-11 |
| KAF8707467.1 | | 191 | 449 | 468 | 7.26e-11 |
| KAF8713575.1 | | 210 | 66 | 80 | 6.47e-04 |
| *Rhizoctonia solani 123e* | | | | | |
| KEP49386.1 | | 152 | 151 | 169 | 6.55e-12 |
| KEP49386.1 | | 191 | 449 | 468 | 7.26e-11 |
| *Rhizoctonia solani AG-1 IA* | | | | | |
| ELU40284.1 | | 210 | 456 | 470 | 6.45e-04 |
| *Rhizoctonia solani AG-1 IB* | | | | | |
| CEL58561.1 | | 152 | 151 | 169 | 6.55e-12 |
| CEL58561.1 | | 191 | 449 | 468 | 7.26e-11 |
| *Rhizoctonia solani AG-3 Rhs1AP* | | | | | |
| EUC60696.1 | | 152 | 151 | 169 | 6.55e-12 |
| EUC60696.1 | | 191 | 449 | 468 | 7.26e-11 |
| *Rhizoctonia solani AG-8 WAC10335* | | | | | |
| KDN46749.1 | | 152 | 65 | 83 | 6.56e-12 |
| KDN34097.1 | | 185 | 219 | 238 | 5.16e-11 |
| KDN43747.1 | | 185 | 97 | 116 | 5.16e-11 |
| KDN46749.1 | | 185 | 79 | 98 | 9.25e-12 |
| KDN46749.1 | | 191 | 363 | 382 | 7.26e-11 |
| *Russula emetica* | | | | | |
| KAF8495713.1 | | 49 | 124 | 133 | 0.91 |
| KAF8494857.1 | | 156 | 471 | 490 | 9.90e-08 |
| KAF8494857.1 | | 158 | 37 | 56 | 3.09e-06 |
| KAF8494857.1 | | 185 | 170 | 189 | 9.24e-12 |
| KAF8492830.1 | | 201 | 11 | 25 | 1.82e-06 |
| KAF8492830.1 | | 210 | 1 | 15 | 8.14e-05 |
| *Sporidiobolus salmonicolor* | | | | | |
| CEQ42946.1 | | 186 | 302 | 320 | 1.10e-06 |
| CEQ42946.1 | | 191 | 331 | 350 | 4.05e-10 |
| *Stachybotrys chartarum IBT 40288* | | | | | |
| KFA76045.1 | | 49 | 379 | 392 | 0.64 |
| *Stachybotrys chartarum IBT 7711* | | | | | |
| KEY69302.1 | | 49 | 379 | 392 | 0.64 |
| *Syncephalastrum racemosum* | | | | | |
| ORY94843.1 | | 169 | 7 | 26 | 4.48e-09 |
| ORY94843.1 | | 191 | 411 | 430 | 4.05e-10 |
| ORY96441.1 | | 222 | 26 | 40 | 0.002 |
| *Thermothelomyces thermophilus ATCC 42464* | | | | | |
| XP_003664039.1 | | 185 | 165 | 184 | 7.26e-11 |
| *Verruconis gallopava* | | | | | |
| XP_016211849.1 | | 165 | 190 | 209 | 5.70e-10 |
| XP_016210414.1 | | 169 | 53 | 72 | 1.60e-09 |
| XP_016210414.1 | | 178 | 367 | 386 | 5.70e-10 |
| *Wallemia mellicola CBS 633.66* | | | | | |
| XP_006958168.1 | | 154 | 190 | 209 | 1.44e-10 |
| *Yarrowia lipolytica* | | | | | |
| KAB8283725.1 | | 164 | 349 | 367 | 3.09e-06 |
| KAB8283725.1 | | 182 | 154 | 173 | 7.27e-11 |
| *Yarrowia lipolytica CLIB122* | | | | | |
| XP_503913.1 | | 164 | 508 | 526 | 3.09e-06 |
| XP_503913.1 | | 182 | 313 | 332 | 7.25e-11 |
