# Supplementary material for: Mining the capacity of human-associated microorganisms to trigger rheumatoid arthritis—A systematic immunoinformatics analysis of T cell epitopes
Source: PLoS One. 2021 Jun 29;16(6):e0253918. doi: 10.1371/journal.pone.0253918 (PMC8241107; doi:10.1371/journal.pone.0253918)
Supplement: S7 Table — (DOCX) [file pone.0253918.s007.docx]

Mining the capacity of human-associated microorganisms to trigger rheumatoid arthritis – a systematic immunoinformatics analysis of T cell epitopes

Jelena Repac^1^, Marija Mandić^1^, Tanja Lunić^1^, Bojan Božić^1*¶^, Biljana Božić Nedeljković^1*¶^

^1^ Institute of Physiology and Biochemistry “Ivan Djaja”, Faculty of Biology, University of Belgrade, Belgrade, Serbia

# **S7 Table. The distribution of BLASTp hits across fungi human pathogen/commensals where the relation between rheumatoid arthritis and the corresponding species has been previously established in literature (PubMed).**

| Fungi | | | | | |
| --- | --- | --- | --- | --- | --- |
| Accession Number | **Epitope Number** | | **Start** | **Stop** | **e value** |
| *Candida albicans* | | | | | |
| KAF6063334.1 | | 154 | 158 | 177 | 4.65e-12 |
| AET14827.1 | | 154 | 158 | 177 | 1.44e-10 |
| *Candida albicans 12C* | | | | | |
| KGT72617.1 | | 154 | 158 | 177 | 4.65e-12 |
| *Candida albicans 19F* | | | | | |
| KGU15991.1 | | 190 | 190 | 209 | 3.30e-12 |
| *Candida albicans Ca6* | | | | | |
| KHC47150.1 | | 190 | 190 | 209 | 3.30e-12 |
| *Candida albicans P34048* | | | | | |
| KGU30551.1 | | 154 | 158 | 177 | 1.44e-10 |
| *Candida albicans P37005* | | | | | |
| KGQ98820.1 | | 154 | 158 | 177 | 4.65e-12 |
| *Candida albicans P57072* | | | | | |
| KGR13197.1 | | 190 | 190 | 209 | 3.30e-12 |
| *Candida albicans P75063* | | | | | |
| KGU36014.1 | | 154 | 158 | 177 | 4.65e-12 |
| *Candida albicans SC5314* | | | | | |
| XP_713669.2 | | 154 | 158 | 177 | 4.65e-12 |
| XP_722186.1 | | 154 | 158 | 177 | 1.44e-10 |
| XP_719462.2 | | 190 | 190 | 209 | 3.30e-12 |
| *Candida albicans WO-1* | | | | | |
| EEQ42742.1 | | 154 | 158 | 177 | 4.65e-12 |
| *Sporothrix schenckii 1099-18* | | | | | |
| XP_016586130.1 | | 4 | 748 | 757 | 1 |
| XP_016592265.1 | | 49 | 767 | 776 | 0.91 |
| *Sporothrix schenckii ATCC 58251* | | | | | |
| ERS98917.1 | | 4 | 732 | 741 | 1 |
| ERS98299.1 | | 49 | 749 | 758 | 0.91 |
