# Supplementary material for: Mining the capacity of human-associated microorganisms to trigger rheumatoid arthritis—A systematic immunoinformatics analysis of T cell epitopes
Source: PLoS One. 2021 Jun 29;16(6):e0253918. doi: 10.1371/journal.pone.0253918 (PMC8241107; doi:10.1371/journal.pone.0253918)
Supplement: S8 Table — (DOCX) [file pone.0253918.s008.docx]

Mining the capacity of human-associated microorganisms to trigger rheumatoid arthritis – a systematic immunoinformatics analysis of T cell epitopes

Jelena Repac^1^, Marija Mandić^1^, Tanja Lunić^1^, Bojan Božić^1*¶^, Biljana Božić Nedeljković^1*¶^

^1^ Institute of Physiology and Biochemistry “Ivan Djaja”, Faculty of Biology, University of Belgrade, Belgrade, Serbia

# **S8 Table. The distribution of BLASTp hits across viruses human pathogen/commensals where the relation between rheumatoid arthritis and the corresponding species has not been previously established in literature (PubMed).**

| Viruses | | | | | |
| --- | --- | --- | --- | --- | --- |
| Accession Number | **Epitope Number** | | **Start** | **Stop** | **e value** |
| *Colobine gammaherpesvirus 1* | | | | | |
| QDQ69216.1 | | 92 | 904 | 913 | 0.88 |
| *Marseillevirus LCMAC101* | | | | | |
| QBK85853.1 | | 152 | 116 | 132 | 1.07e-06 |
| QBK85853.1 | | 154 | 163 | 182 | 1.36e-07 |
| QBK85853.1 | | 158 | 6 | 16 | 0.37 |
| QBK85853.1 | | 165 | 196 | 214 | 3.44e-08 |
| QBK85853.1 | | 166 | 465 | 483 | 7.83e-10 |
| QBK85853.1 | | 180 | 452 | 469 | 6.84e-08 |
| *Marseillevirus LCMAC201* | | | | | |
| QBK87512.1 | | 154 | 159 | 178 | 2.44e-08 |
| QBK87512.1 | | 156 | 432 | 451 | 7.06e-11 |
| QBK87512.1 | | 158 | 5 | 16 | 0.13 |
| QBK87512.1 | | 159 | 27 | 45 | 7.06e-11 |
| QBK87512.1 | | 161 | 174 | 194 | 2.71e-07 |
| QBK87512.1 | | 165 | 191 | 209 | 6.68e-05 |
| QBK87512.1 | | 166 | 462 | 480 | 1.98e-10 |
| QBK87512.1 | | 167 | 355 | 374 | 1.07e-06 |
| QBK87512.1 | | 169 | 12 | 31 | 2.44e-08 |
| QBK87512.1 | | 178 | 327 | 344 | 5.39e-07 |
| QBK87512.1 | | 180 | 447 | 466 | 1.07e-06 |
| *Marseillevirus LCMAC202* | | | | | |
| QBK87811.1 | | 154 | 159 | 178 | 7.83e-10 |
| QBK87831.1 | | 154 | 160 | 179 | 5.55e-10 |
| QBK87831.1 | | 156 | 433 | 452 | 2.79e-10 |
| QBK87844.1 | | 158 | 7 | 16 | 0.73 |
| QBK87811.1 | | 159 | 26 | 44 | 2.44e-08 |
| QBK87844.1 | | 159 | 27 | 45 | 1.92e-07 |
| QBK87831.1 | | 161 | 175 | 195 | 1.22e-08 |
| QBK87831.1 | | 163 | 268 | 285 | 9.42e-05 |
| QBK87831.1 | | 166 | 463 | 481 | 6.16e-09 |
| QBK87831.1 | | 167 | 356 | 375 | 5.39e-07 |
| QBK87831.1 | | 169 | 12 | 31 | 7.83e-10 |
| QBK87811.1 | | 176 | 55 | 74 | 5.39e-07 |
| QBK87831.1 | | 176 | 56 | 75 | 3.82e-07 |
| QBK87811.1 | | 178 | 330 | 344 | 3.01e-06 |
| QBK87831.1 | | 178 | 328 | 345 | 7.60e-07 |
| QBK87831.1 | | 180 | 448 | 467 | 4.25e-06 |
