# Supplementary material for: Mining the capacity of human-associated microorganisms to trigger rheumatoid arthritis—A systematic immunoinformatics analysis of T cell epitopes
Source: PLoS One. 2021 Jun 29;16(6):e0253918. doi: 10.1371/journal.pone.0253918 (PMC8241107; doi:10.1371/journal.pone.0253918)
Supplement: S9 Table — (DOCX) [file pone.0253918.s009.docx]

Mining the capacity of human-associated microorganisms to trigger rheumatoid arthritis – a systematic immunoinformatics analysis of T cell epitopes

Jelena Repac^1^, Marija Mandić^1^, Tanja Lunić^1^, Bojan Božić^1*¶^, Biljana Božić Nedeljković^1*¶^

^1^ Institute of Physiology and Biochemistry “Ivan Djaja”, Faculty of Biology, University of Belgrade, Belgrade, Serbia

# **S9 Table.** HLA alleles associated with Rheumatoid Arthritis.

| **No**. | **HLA alleles** | **RA association** | **Reference** |
| --- | --- | --- | --- |
| 1 | *DRB1*01:01* | Well documented | [[1](#_ENREF_1)] |
| 2 | *DRB1*03:01* | Associated with anti-CCP-negative arthritis  *protective in Asians | [[2](#_ENREF_2)]  [[3](#_ENREF_3)] |
| 3 | *DRB1*04:01* | Well documented | [[1](#_ENREF_1), [4](#_ENREF_4)] |
| 4 | *DRB1*04:05* | Well documented | [[1](#_ENREF_1)] |
| 5 | *DRB1*07:01* | Protective effect against ACPA-Positive RA in a Latin American Admixed Population | [[5](#_ENREF_5)] |
| 6 | *DRB1*08:02* | Protective effect against ACPA-Positive RA in a Latin American Admixed Population | [[5](#_ENREF_5)] |
| 7 | *DRB1*09:01* | Increased in ACPA-positive RA patients | [[5](#_ENREF_5)] |
| 8 | *DRB1*11:01* | Associated with juvenile idiopathic arthritis | [[6](#_ENREF_6)] |
| 9 | *DRB1*12:01* | Associated for ACPA-negative RA | [[7](#_ENREF_7)] |
| 10 | *DRB1*13:02* | Associated with susceptibility to RA  Specific HLA-DRB1 alleles predispose to RA while others encoding the sequence DERAA are RA-protective | [[1](#_ENREF_1)]  [[8](#_ENREF_8)] |
| 11 | *DRB1*15:01* | Associated with  susceptibility to RA | [[1](#_ENREF_1)] |
| 12 | *DRB3*01:01* | HLA-DRB3⁄ 03:01 was significantly lower in RA patients | [[9](#_ENREF_9)] |
| 13 | *DRB3*02:02* | HLA-DRB3⁄ 03:01 was significantly lower in RA patients | [[9](#_ENREF_9)] |
| 14 | *DRB4*01:01* | Associated with susceptibility to RA | [[10](#_ENREF_10)] |
| 15 | *DRB5*01:01* | Not found | / |
| 16 | *DPA1*01/****DPB1*04:01*** | Associated with susceptibility to RA | [[11](#_ENREF_11)] |
| 17 | *DPA1*01:03/****DPB1*02:01*** | DPB1*0201 was higher in seronegative RA patients. | [[12](#_ENREF_12)] |
| 18 | *DPA1*02:01/****DPB1*01:01*** | Negatively associated | [[11](#_ENREF_11)] |
| 19 | *DPA1*02:01/****DPB1*05:01*** | Negatively associated | [[11](#_ENREF_11)] |
| 20 | *DPA1*03:01/****DPB1*04:02*** | Negatively associated | [[11](#_ENREF_11)] |
| 21 | *DQA1*01:01/DQB1*05:01* | Not found | / |
| 22 | *DQA1*01:02/****DQB1*06:02*** | Negatively associated | [[13](#_ENREF_13)] |
| 23 | ***DQA1*03:01****/DQB1*03:02* | Associated with susceptibility to RA | [[10](#_ENREF_10)] |
| 24 | *DQA1*04:01/DQB1*04:02* | Not found | / |
| 25 | *DQA1*05:01/****DQB1*02:01*** | Negatively associated | [[13](#_ENREF_13)] |
| 26 | *DQA1*05:01/****DQB1*03:01*** | Associated with susceptibility to RA | [[10](#_ENREF_10)] |

# **SI_References**

1. Karami J, Aslani S, Jamshidi A, Garshasbi M, Mahmoudi M. Genetic implications in the pathogenesis of rheumatoid arthritis; an updated review. Gene. 2019;702:8-16.

2. Verpoort KN, van Gaalen FA, van der Helm‐van Mil AH, Schreuder GM, Breedveld FC, Huizinga TW, et al. Association of HLA–DR3 with anti–cyclic citrullinated peptide antibody–negative rheumatoid arthritis. Arthritis & Rheumatism: Official Journal of the American College of Rheumatology. 2005;52(10):3058-62.

3. Wysocki T, Olesińska M, Paradowska-Gorycka A. Current Understanding of an Emerging Role of HLA-DRB1 Gene in Rheumatoid Arthritis–From Research to Clinical Practice. Cells. 2020;9(5):1127.

4. Balandraud N, Picard C, Reviron D, Landais C, Toussirot E, Lambert N, et al. HLA-DRB1 genotypes and the risk of developing anti citrullinated protein antibody (ACPA) positive rheumatoid arthritis. PLoS One. 2013;8(5):e64108.

5. Castro-Santos P, Olloquequi J, Verdugo RA, Gutiérrez MA, Pinochet C, Quiñones LA, et al. HLA-DRB1* 07: 01 and* 08: 02 alleles confer a protective effect against ACPA-positive rheumatoid arthritis in a latin american admixed population. Biology. 2020;9(12):467.

6. Ombrello MJ, Remmers EF, Tachmazidou I, Grom A, Foell D, Haas J-P, et al. HLA-DRB1* 11 and variants of the MHC class II locus are strong risk factors for systemic juvenile idiopathic arthritis. Proceedings of the National Academy of Sciences. 2015;112(52):15970-5.

7. Terao C, Ohmura K, Kochi Y, Ikari K, Maruya E, Katayama M, et al. A large-scale association study identified multiple HLA-DRB1 alleles associated with ACPA-negative rheumatoid arthritis in Japanese subjects. Annals of the rheumatic diseases. 2011;70(12):2134-9.

8. Kanaan SB, Sensoy O, Yan Z, Gadi VK, Richardson ML, Nelson JL. Immunogenicity of a rheumatoid arthritis protective sequence when acquired through microchimerism. Proceedings of the National Academy of Sciences. 2019;116(39):19600-8.

9. Louthrenoo W, Kasitanon N, Wangkaew S, Kuwata S, Takeuchi F. Distribution of HLA-DR alleles among Thai patients with rheumatoid arthritis. Human immunology. 2015;76(2-3):113-7.

10. Morling N, Andersen V, Fugger L, Georgsen J, Halberg P, Oxholm P, et al. Immunogenetics of rheumatoid arthritis and primary Sjögren's syndrome: DNA polymorphism of HLA class II genes. Disease markers. 1991;9(5):289-96.

11. Jiang L, Jiang D, Han Y, Shi X, Ren C. Association of HLA-DPB1 polymorphisms with rheumatoid arthritis: A systemic review and meta-analysis. International Journal of Surgery. 2018;52:98-104.

12. Singal D, Sastry A, Buchanan W. HLA-DPB1 alleles in patients with rheumatoid arthritis. Clinical and experimental rheumatology. 1994;12(2):183-6.

13. Wu J, Li J, Li S, Zhang T-P, Li L-J, Lv T-T, et al. Association of HLA-DQB1 polymorphisms with rheumatoid arthritis: a meta-analysis. Postgraduate medical journal. 2017;93(1104):618-25.
