# Supplementary material for: Mining the capacity of human-associated microorganisms to trigger rheumatoid arthritis—A systematic immunoinformatics analysis of T cell epitopes
Source: PLoS One. 2021 Jun 29;16(6):e0253918. doi: 10.1371/journal.pone.0253918 (PMC8241107; doi:10.1371/journal.pone.0253918)
Supplement: S10 Table — (DOCX) [file pone.0253918.s010.docx]

Mining the capacity of human-associated microorganisms to trigger rheumatoid arthritis – a systematic immunoinformatics analysis of T cell epitopes

Jelena Repac^1^, Marija Mandić^1^, Tanja Lunić^1^, Bojan Božić^1*¶^, Biljana Božić Nedeljković^1*¶^

^1^ Institute of Physiology and Biochemistry “Ivan Djaja”, Faculty of Biology, University of Belgrade, Belgrade, Serbia

# **S10 Table**. **A comprehensive list (homologues to the IEDB experiential and newly predicted epitopes) rheumatoid arthritis triggering epitopes homologous to Antigen_1 (endoplasmic reticulum chaperone BiP).**

| No. | Experimental | Putative/Predicted | | | |
| --- | --- | --- | --- | --- | --- |
|  |  | **Bacteria** | | **Fungi** | |
|  |  | **No_PubMed** | **PubMed** | **No_PubMed** | **PubMed** |
| 1 | APEEISAMVLTKMKETAEAY | NTFYAVKRLIGRRYD | EAVAYGAAVQAAILS | IINEPTAAAIAYGLD | EAVAYGAAVQAAILS |
| 2 | ATNGDTHLGGEDFDQRVMEH | DEVVAIGAAIQGAVL | EAVAYGAAVQAAILS | DKVLLLDVNPLTLGI | IINEPTAAAIAYGLD |
| 3 | DAGTIAGLNVMRIINEPTAA | PQIEVTFDIDANGIV | DYSVTVSRARFEELC | NKLGKFELNGIAPAP | DLLLLDVAPLSLGIE |
| 4 | DIKFLPFKVVEKKTKPYIQV | DVKDVLLLDVTPLSL | IINEPTAAAIAYGLD | PQIEVTFEIDANGIL | EAVAYGAAVQAAILS |
| 5 | DNQPTVTIKVYEGERPLTKD | IINEPTAAALAYGME | IINEPTAAAIAYGLD | IINEPTAAAIAYGLD | VTFSLDANGILKVEA |
| 6 | DVSLLTIDNGVFEVVATNGD | DEVVALGASLQAGVL | DEVVAVGAAIQGAIL | DKVLLLDVNPLTLGI | DEAVAYGAAVQAGVL |
| 7 | EDKKEDVGTVVGIDLGTTYS | PQIEVTFDIDANGIV | LNVQRIVNEPTAAAL | NKLGKFELNGIAPAP | IVNEPTAAAIAYGLD |
| 8 | EIIANDQGNRITPSYVAFTP | EVKDVLLLDVTPLSL | IVNEPTAAALAYGVD | PQIEVTFEIDANGIL | VMTTLIKRNTAIPTK |
| 9 | EKFAEEDKKLKERIDTRNEL | IVNEPTAAALAYGLD | LNVRRIVNEPTAAAL | IVNEPTAAAIAYGLD | VTFSLDANGILKVEA |
| 10 | EPTAAAIAYGLDKREGEKNI | DVKDVLLLDVTPLSL | IVNEPTAAALAYGVD | PQIEVTFEIDANGLL | DEAVAYGAAVQAGVL |
| 11 | ESHQDADIEDFKAKKKELEE | IINEPTAAALAYGMD | LNVRRIVNEPTAAAL | FSETLTRSKFEELNM | IVNEPTAAAIAYGLD |
| 12 | EVEKAKRALSSQHQARIEIE | DEVVALGAAVQAAII | DEVVAVGAAIQGAIL | IINEPTAAAIAYGLD | VMTTLIKRNTAIPTK |
| 13 | FEIDVNGILRVTAEDKGTGN | DVKDVLLLDVTPLSL | IVNEPTAAALAYGVD | DKVLLLDVNPLTLGI | EAVAYGAAVQAAILS |
| 14 | GEKNILVFDLGGGTFDVSLL | IINEPTAAALAYGMD | DEVVAVGAAIQGAIL | VTFEIDANGILKVSA | IINEPTAAAIAYGLD |
| 15 | GIPPAPRGVPQIEVTFEIDV | GRRDLLLLDVVPLSL | LNVQRIVNEPTAAAL | SSIVLMDVNPLTLGI | DLLLLDVAPLSLGIE |
| 16 | GKEPSRGINPDEAVAYGAAV | KLIHRNSTVPATATT | PQEISAMILQKMKKT | VSFELDANGILKVSA | EAVAYGAAVQAAILS |
| 17 | GSAGPPPTGEEDTAELHHHH | LDVLRIVNEPTAAAL | IVNEPTAAALAYGVD | RVVNEPTAAALAYGL | DEAVAYGAAVQAAIL |
| 18 | GTTYSCVGVFKNGRVEIIAN | DGVFKVLATNGDTYL | PQIEVTFDIDANGIV | SGIVLMDVNPLTLGI | IINEPTAAAIAYGLD |
| 19 | IETVGGVMTKLIPRNTVVPT | IVNEPTAAALAYGLD | IINEPTAAALAYGAD | VSFELDANGILKVSA | SKTQDILLLDVAPLS |
| 20 | IGDKEKLGGKLSSEDKETME | IINEPTAAALAYGLD | GDVKGLLLLDVTPLS | RVVNEPTAAALAYGL | DEAVAYGAAVQAAIL |
| 21 | KDVRKDNRAVQKLRREVEKA | LNVSVTLTREKFEEL |  | SGIVLMDVNPLTLGI | EAVAYGAAVQAAILS |
| 22 | KELEEIVQPIISKLYGSAGP | EVVAQGAAIQGAILQ |  | VSFELDANGILKVSA | IINEPTAAAIAYGLD |
| 23 | KETMEKAVEEKIEWLESHQD | PLEVIFEYDSDGIIH |  | RVVNEPTAAALAYGL | DLLLLDVAPLSLGIE |
| 24 | KGTGNKNKITITNDQNRLTP | IINEPTAAALAYGLD |  | GKVEIFVNDQGNRIT | EAVAYGAAVQAAILS |
| 25 | KNQLTSNPENTVFDAKRLIG | VMEHFIKLYKKKKGK |  | VNEPTAAAIAYGLDK | VTFSLDANGILKVEA |
| 26 | KRLIGRTWNDPSVQQDIKFL | DFDQRVMEHFIKLYK |  | IDRMVAEAAEFAEED | DEAVAYGAAVQAGVL |
| 27 | LKKSDIDEIVLVGGSTRIPK | DEAVAYGAAVQAGVL |  | SEIVLMDVNPLTLGI | IVNEPTAAAIAYGLD |
| 28 | NRLTPEEIERMVNDAEKFAE | IINEPTAAAIAYGLD |  | VSFELDANGILKVTA | VMTTLIKRNTAIPTK |
| 29 | PLTKDNHLLGTFDLTGIPPA | EDVVQPIIAKLYQGQ |  | GKVEIFVNDQGNRIT | EAVAYGAAVQAAILS |
| 30 | PYIQVDIGGGQTKTFAPEEI | PQIEVTFEIDANGIL |  | VNEPTAAAIAYGLDK | IINEPTAAAIAYGLD |
| 31 | RIEIESFYEGEDFSETLTRA | DAIVLLDVNPLTMGI |  | IDRMVAEAAEFAEED | DLLLLDVAPLSLGIE |
| 32 | RSTMKPVQKVLEDSDLKKSD | DECVAIGAAIQAAVL |  | SEIVLMDVNPLTLGI | EAVAYGAAVQAAILS |
| 33 | RVMEHFIKLYKKKTGKDVRK | DECVAIGAAIQAAVL |  | VSFELDANGILKVTA | VTFSLDANGILKVEA |
| 34 | TAEAYLGKKVTHAVVTVPAY | PQIEVTFDIDANGIV |  | GKVEIFVNDQGNRIT | DEAVAYGAAVQAGVL |
| 35 | TGDLVLLDVCPLTLGIETVG | GDVKGLLLLDVTPLS |  | VNEPTAAAIAYGLDK | IVNEPTAAAIAYGLD |
| 36 | TLTRAKFEELNMDLFRSTMK | LEVMRIINEPTAASL |  | SEIVLMDVNPLTLGI | VMTTLIKRNTAIPTK |
| 37 | TRIPKIQQLVKEFFNGKEPS | PQEISAMIVQKLKAD |  | VSFELDANGILKVTA | EAVAYGAAVQAAILS |
| 38 | TRNELESYAYSLKNQIGDKE | VEDVLLLDVTPLSLG |  | IVNEPTAAAIAYGLD | IINEPTAAAIAYGLD |
| 39 | TVPAYFNDAQRQATKDAGTI | IINEPTAAALAYGLD |  | AEVVLMDVNPLTMGI | DLLLLDVAPLSLGIE |
| 40 | TVVPTKKSQIFSTASDNQPT | PQEISAMIVQKLKAD |  | IDRMVAEAEEFAEED | EAVAYGAAVQAAILS |
| 41 | VAFTPEGERLIGDAAKNQLT | VEDVLLLDVTPLSLG |  | TRTIFDIKRLIGRKW |  |
| 42 | YGAAVQAGVLSGDQDTGDLV | IINEPTAAALAYGLD |  | IVNEPTAAAIAYGLD |  |
| 43 |  | VEDVLLLDVTPLSLG |  | GTSEVVLMDVNPLTL |  |
| 44 |  | IINEPTAAALAYGLD |  | IDRMVAEAEEFAEED |  |
| 45 |  | DESVAIGAAVQAGVL |  | TRTIFDIKRLIGRKW |  |
| 46 |  | VTFDIDANGIVHVSA |  | EDLILMDVNPLTLGI |  |
| 47 |  | EVKDVLLLDVTPLSL |  | VNEPTAAAIAYGLDK |  |
| 48 |  | PQEISAMVLQKLKQD |  | GKVEIIVNDQGNRIT |  |
| 49 |  | DESVAIGAAVQAGVL |  | IDRMVAEAEEFAEAD |  |
| 50 |  | IINEPTAAALAYGLD |  | VSFELDANGILKVSA |  |
| 51 |  | DESVAIGAAVQAGVL |  | EDLILMDVNPLTLGI |  |
| 52 |  | VTFDIDANGIVHVSA |  | EDLILMDVNPLTLGI |  |
| 53 |  | EVKDVLLLDVTPLSL |  | GKVEIIVNDQGNRIT |  |
| 54 |  | PQEISAMVLQKLKQD |  | DEAVAYGAAVQAGIL |  |
| 55 |  | DESVAIGAAVQAGVL |  | IDRMVAEAEEFAEAD |  |
| 56 |  | IINEPTAAALAYGLD |  | VSFELDANGILKVTA |  |
| 57 |  | ERMQQEAEVFAEEDR |  | RVVNEPTAAALAYGL |  |
| 58 |  | NLLLLDITPLSLGIE |  | EDLILMDVNPLTLGI |  |
| 59 |  | PQEISAMVLQKLKQD |  | EDLILMDVNPLTLGI |  |
| 60 |  | DEAVGLGAAIQAGVL |  | VNEPTAAAIAYGLDK |  |
| 61 |  | VKDVVLLDVTPLSLG |  | GKVEIIVNDQGNRIT |  |
| 62 |  | IINEPTAAALAYGLD |  | IDRMVAEAEEFAEAD |  |
| 63 |  | PQIEVTFDIDANGIV |  | VSFELDANGILKVSA |  |
| 64 |  | DVLLIDVTPLSLGIE |  | EDLILMDVNPLTLGI |  |
| 65 |  | IINEPTAAALAYGLE |  | EAVAYGAAVQAAILS |  |
| 66 |  | KDVLLLDVTPLSLGI |  | IINEPTAAAIAYGLD |  |
| 67 |  | EMQFEVLSTNGDTFL |  | CVGIYRDDRIEIIAN |  |
| 68 |  | KDVLLLDVTPLSLGI |  | EILLLDVAPLSLGIE |  |
| 69 |  | EMQFEVLSTNGDTFL |  | EAVAYGAAVQAAILS |  |
| 70 |  | PQIEVTFDIDANGIV |  | DEAVAFGAAVQAGVL |  |
| 71 |  | LNVQRIVNEPTAAAL |  | IVNEPTAAAIAYGLD |  |
| 72 |  | KDVLLIDVTPLSLGI |  | EEIVLMDVNPLTLGI |  |
| 73 |  | IVNEPTAAALAYGLE |  | VSFELDANGILKVSA |  |
| 74 |  | DEVVALGAAIQAGVL |  | RNTIFDIKRMIGQKF |  |
| 75 |  | EVKDILLLDVTPLSL |  | DEAVAFGAAVQAGVL |  |
| 76 |  | NKSLGTFRLDGIPPA |  | IVNEPTAAAIAYGLD |  |
| 77 |  | IEVKRIINEPTAASL |  | EDIVLMDVNPLTLGI |  |
| 78 |  | DEVVAVGAAIQGAIL |  | LNVLRIVNEPTAAAI |  |
| 79 |  | LNVQRIVNEPTAAAL |  | VSFELDANGILKVSA |  |
| 80 |  | PQEISAMILQKMKKT |  | ITPSYVAFTEEERLV |  |
| 81 |  | IVNEPTAAALAYGVD |  | IINEPTAAAIAYGLD |  |
| 82 |  | IINEPTAAALAYGID |  | DEAVAYGAAVQAGIL |  |
| 83 |  | PKIEVTFDIDANGIV |  | DLLLLDVNPLTLGIE |  |
| 84 |  | DVLLIDVTPLSLGIE |  | PQIEVTFEIDANGIM |  |
| 85 |  | IINEPTAAALAYGLE |  | DEAVAYGAAVQAAIL |  |
| 86 |  | PQIEVTFDIDANGIV |  | IINEPTAAAIAYGLD |  |
| 87 |  | DVLLIDVTPLSLGIE |  | DLLLLDVAPLSLGIE |  |
| 88 |  | IINEPTAAALAYGLE |  | DEAVAYGAAVQAAIL |  |
| 89 |  | TPSMVAFRADGEVLV |  | IERMVAEAEMFAEED |  |
| 90 |  | DEVVALGAAIQAGVL |  | VNEPTAAAIAYGLDK |  |
| 91 |  | QVEDVLLLDVAPLSL |  | GKVEIIVNDQGNRIT |  |
| 92 |  | IINEPTAAALAYGLE |  | ADIVLMDVNPLTLGI |  |
| 93 |  | DEVVAQGAAIQGAIM |  | VSFELDANGILRVTA |  |
| 94 |  | PLEVIFEYDSDGIIH |  | DQRVSDYFVKLYNKK |  |
| 95 |  | IINEPTAAALAYGLD |  | GKVEIFVNDQGNRIT |  |
| 96 |  |  |  | VNEPTAAAIAYGLDK |  |
| 97 |  |  |  | IDRMVAEAAEFAEED |  |
| 98 |  |  |  | SEIVLMDVNPLTLGI |  |
| 99 |  |  |  | VSFELDANGILKVTA |  |
| 100 |  |  |  | GKVEIFVNDQGNRIT |  |
| 101 |  |  |  | KNQFAANPTRTIYDV |  |
| 102 |  |  |  | VNEPTAAAIAYGLDK |  |
| 103 |  |  |  | IDRMVAEAAEFAEED |  |
| 104 |  |  |  | SEIVLMDVNPLTLGI |  |
| 105 |  |  |  | VSFELDANGILKVTA |  |
| 106 |  |  |  | GKVEIFVNDQGNRIT |  |
| 107 |  |  |  | KNQFAANPTRTIYDV |  |
| 108 |  |  |  | VNEPTAAAIAYGLDK |  |
| 109 |  |  |  | IDRMVAEAAEFAEED |  |
| 110 |  |  |  | SEIVLMDVNPLTLGI |  |
| 111 |  |  |  | VSFELDANGILKVTA |  |
| 112 |  |  |  | GKVEIFVNDQGNRIT |  |
| 113 |  |  |  | VNEPTAAAIAYGLDK |  |
| 114 |  |  |  | IDRMVAEAAEFAEED |  |
| 115 |  |  |  | SEIVLMDVNPLTLGI |  |
| 116 |  |  |  | VSFELDANGILKVTA |  |
| 117 |  |  |  | INEPTAAAIAYGLNK |  |
| 118 |  |  |  | PQIEVTFEIDANGIM |  |
| 119 |  |  |  | KDVVLMDVNPLTLGI |  |
| 120 |  |  |  | VTFELDANGILRVSA |  |
| 121 |  |  |  | RVVNEPTAAALAYGL |  |
| 122 |  |  |  | ITPSWVAFTEEERLI |  |
| 123 |  |  |  | KTEWLEENPAAEAED |  |
| 124 |  |  |  | IVNEPTAAAIAYGLD |  |
| 125 |  |  |  | LTVLRIVNEPTAAAI |  |
| 126 |  |  |  | VTFEIDANGILKVSA |  |
| 127 |  |  |  | GKVEIIANDQGNRIT |  |
| 128 |  |  |  | SSVVLMDVNPLTLGI |  |
| 129 |  |  |  | GKVDIIVNDQGNRIT |  |
| 130 |  |  |  | VSFELDANGILKVSA |  |
| 131 |  |  |  | RVVNEPTAAALAYGL |  |
| 132 |  |  |  | IINEPTAAAIAYGLD |  |
| 133 |  |  |  | DKVLLLDVNPLTLGI |  |
| 134 |  |  |  | PQIEVTFEVDANGIL |  |
| 135 |  |  |  | IDRMVQEAEEFAEED |  |
| 136 |  |  |  | IINEPTAAAIAYGLD |  |
| 137 |  |  |  | DKVLLLDVNPLTLGI |  |
| 138 |  |  |  | PQIEVTFEIDANGIL |  |
| 139 |  |  |  | IDRMVQEAEEFAEED |  |
| 140 |  |  |  | VTFSLDANGILKVEA |  |
| 141 |  |  |  | DEAVAYGAAVQAGVL |  |
| 142 |  |  |  | IVNEPTAAAIAYGLD |  |
| 143 |  |  |  | VMTTLIKRNTAIPTK |  |
| 144 |  |  |  | GKVEIIVNDQGNRIT |  |
| 145 |  |  |  | GSLVLMDVNPLTLGI |  |
| 146 |  |  |  | AAKNQFSSNPARTIF |  |
| 147 |  |  |  | VSFELDANGILKVSA |  |
| 148 |  |  |  | RVVNEPTAAALAYGL |  |
| 149 |  |  |  | EAVAYGAAVQAAILS |  |
| 150 |  |  |  | INEPTAAAIAYGFDK |  |
| 151 |  |  |  | SILLLDVAPLSLGIE |  |
| 152 |  |  |  | EAVAYGAAVQAAILS |  |
| 153 |  |  |  | EAVAYGAAVQAAILS |  |
| 154 |  |  |  | INEPTAAAIAYGFDK |  |
| 155 |  |  |  | SILLLDVAPLSLGIE |  |
| 156 |  |  |  | EAVAYGAAVQAAILS |  |
| 157 |  |  |  | EAVAYGAAVQAAILS |  |
| 158 |  |  |  | INEPTAAAIAYGFDK |  |
| 159 |  |  |  | SILLLDVAPLSLGIE |  |
| 160 |  |  |  | EAVAYGAAVQAAILS |  |
| 161 |  |  |  | VNEPTAAAIAYGLDK |  |
| 162 |  |  |  | GHVEIILNDQGNRIT |  |
| 163 |  |  |  | VSFELDANGILKVSA |  |
| 164 |  |  |  | EELLLMDVNPLTLGI |  |
| 165 |  |  |  | DEAVAFGAAVQAGVL |  |
| 166 |  |  |  | VNEPTAAAIAYGLDM |  |
| 167 |  |  |  | IDRMVAEAAEFAEED |  |
| 168 |  |  |  | EDVVLMDVNPLTLGI |  |
| 169 |  |  |  | DEAVAYGAAVQAGVL |  |
| 170 |  |  |  | IVNEPTAAAIAYGLD |  |
| 171 |  |  |  | VTFALDANGILKVEA |  |
| 172 |  |  |  | VMTTLIKRNTAIPTK |  |
| 173 |  |  |  | RITPSYVAFTPERLV |  |
| 174 |  |  |  | IVNEPTAAAIAYGLD |  |
| 175 |  |  |  | TPEEVSAMVLQKMKE |  |
| 176 |  |  |  | ERMVAEAEEFAEQDE |  |
| 177 |  |  |  | RITPSYVAFTPERLV |  |
| 178 |  |  |  | IVNEPTAAAIAYGLD |  |
| 179 |  |  |  | TPEEVSAMVLQKMKE |  |
| 180 |  |  |  | ERMVAEAEEFAEQDE |  |
| 181 |  |  |  | RITPSYVAFTPERLV |  |
| 182 |  |  |  | IVNEPTAAAIAYGLD |  |
| 183 |  |  |  | TPEEVSAMVLQKMKE |  |
| 184 |  |  |  | ERMVAEAEEFAEQDE |  |
| 185 |  |  |  | DEAVAFGAAVQAGVL |  |
| 186 |  |  |  | VNEPTAAAIAYGLDM |  |
| 187 |  |  |  | IDRMVAEAAEFAEED |  |
| 188 |  |  |  | EDVVLMDVNPLTLGI |  |
| 189 |  |  |  | PQIEVSFELDPNGIL |  |
| 190 |  |  |  | DEAVAFGAAVQAGVL |  |
| 191 |  |  |  | IVNEPTAAAIAYGLD |  |
| 192 |  |  |  | EEIVLMDVNPLTLGI |  |
| 193 |  |  |  | VSFELDANGILKVSA |  |
| 194 |  |  |  | ITPSYVAFTEEERLV |  |
| 195 |  |  |  | KDLVLMDVNPLTLGI |  |
| 196 |  |  |  | IVNEPTAAALAYGLD |  |
| 197 |  |  |  | DEAVAFGAAVQAGVL |  |
| 198 |  |  |  | IVNEPTAAAIAYGLD |  |
| 199 |  |  |  | EEIVLMDVNPLTLGI |  |
| 200 |  |  |  | LNVLRIVNEPTAAAI |  |
| 201 |  |  |  | VSFELDANGILKVSA |  |
| 202 |  |  |  | VNEPTAAAIAYGLDK |  |
| 203 |  |  |  | GKVEIIVNDQGNRIT |  |
| 204 |  |  |  | EDIVLMDVNPLTLGI |  |
| 205 |  |  |  | VSFELDANGILKVSA |  |
| 206 |  |  |  | AGTIAGLTVLCIVNK |  |
| 207 |  |  |  | FSETLTRSKFEELNI |  |
| 208 |  |  |  | QIEVTFEIDGNGILK |  |
| 209 |  |  |  | IVNEPTAAAIAYGLD |  |
| 210 |  |  |  | LTVLRIVNEPTAAAI |  |
| 211 |  |  |  | PQIEVTFEIDANGIL |  |
| 212 |  |  |  | FSETLTRSKFEELNI |  |
| 213 |  |  |  | IVNEPTAAAIAYGLD |  |
| 214 |  |  |  | AEAYLGNKVTHAVVT |  |
| 215 |  |  |  | LTVLRIVNEPTAAAI |  |
| 216 |  |  |  | VTFEIDANGILKVAA |  |
| 217 |  |  |  | FSETLTRSKFEELNI |  |
| 218 |  |  |  | IVNEPTAAAIAYGLD |  |
| 219 |  |  |  | LTILRIVNEPTAAAI |  |
| 220 |  |  |  | AEAYLGNKVTHAVVT |  |
| 221 |  |  |  | PQIEVTFEIDANGIL |  |
| 222 |  |  |  | AGTIAGLTILRIVNE |  |
| 223 |  |  |  | FSETLTRSKFEELNI |  |
| 224 |  |  |  | IVNEPTAAAIAYGLD |  |
| 225 |  |  |  | AEAYLGNKVTHAVVT |  |
| 226 |  |  |  | LTVLRIVNEPTAAAI |  |
| 227 |  |  |  | VTFEIDANGILKVAA |  |
| 228 |  |  |  | FSETLTRSKFEELNI |  |
| 229 |  |  |  | SAKNAFHSNPTNTVF |  |
| 230 |  |  |  | IINEPTAAAIAYGLD |  |
| 231 |  |  |  | DNRLIEYFVKQYKKK |  |
| 232 |  |  |  | AGTIAGLQILRIINE |  |
